# Contribution of a C-Terminal Extension to the Substrate Affinity and Oligomeric Stability of Aldehyde Dehydrogenase from *Thermus thermophilus* HB27

**DOI:** 10.1021/acs.biochem.3c00698

**Published:** 2024-04-11

**Authors:** Wiktoria Brytan, Kim Shortall, Francisco Duarte, Tewfik Soulimane, Luis Padrela

**Affiliations:** † Department of Chemical Sciences, Bernal Institute, University of Limerick, Limerick V94 T9PX, Ireland; ‡ SSPC − The Science Foundation Ireland Research Centre for Pharmaceuticals, Limerick V94 T9PX,Ireland

## Abstract

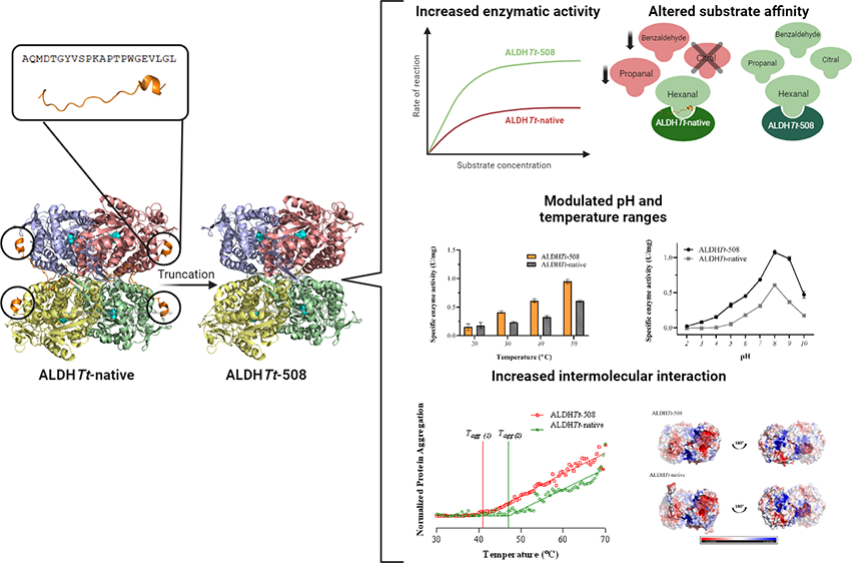

Aldehyde dehydrogenase enzymes (ALDHs) are widely studied for their roles in disease propagation and cell metabolism. Their use in biocatalysis applications, for the conversion of aldehydes to carboxylic acids, has also been recognized. Understanding the structural features and functions of both prokaryotic and eukaryotic ALDHs is key to uncovering novel applications of the enzyme and probing its role in disease propagation. The thermostable enzyme ALDH*Tt* originating from*Thermus thermophilus*, strain HB27, possesses a unique extension of its C-terminus, which has been evolutionarily excluded from mesophilic counterparts and other thermophilic enzymes in the same genus. In this work, the thermophilic adaptation is studied by the expression and optimized purification of mutant ALDH*Tt-*508, with a 22-amino acid truncation of the C-terminus. The mutant shows increased activity throughout production compared to native ALDH*Tt*, indicating an opening of the active site upon C-terminus truncation and giving rationale into the evolutionary exclusion of the C-terminal extension from similar thermophilic and mesophilic ALDH proteins. Additionally, the C-terminus is shown to play a role in controlling substrate specificity of native ALDH, particularly in excluding catalysis of certain large and certain aromatic ortho-substituted aldehydes, as well as modulating the protein’s pH tolerance by increasing surface charge. Dynamic light scattering and size-exclusion HPLC methods are used to show the role of the C-terminus in ALDH*Tt* oligomeric stability at the cost of catalytic efficiency. Studying the aggregation rate of ALDH*Tt* with and without a C-terminal extension leads to the conclusion that ALDH*Tt* follows a monomolecular reaction aggregation mechanism.

## Introduction

Thermophiles are bacteria that are abundant in versatility, living in extreme temperature environments such as volcanoes, hot springs, and deep-sea thermal vents. Proteins expressed by thermophilic bacteria have been applied in industry and research, from food production and detergents to genetic engineering applications.^1^ The mechanisms by which these proteins withstand high temperatures, often exceeding 100 °C, are less well-known. The thermodynamic equilibria between the folded and denatured states are kept in balance by a range of evolutionary adaptations including, but not limited to, increased hydrophobicity of residues and resulting secondary structures, increased number of disulfide bonds, and formation of salt bridges between residues.^2^ Additionally, thermophilic enzymes tend to form larger oligomeric states, indicating the role of quaternary structure in their stability.^3^ Due to a multitude of mechanisms by which these proteins survive, it is often not possible to identify specific structural features that can be studied in depth.

C-Terminus adaptations found in some thermophilic proteins have been recognized as such structural features, or stability “tags”, as they have a major impact on overall protein thermostability and oligomeric state. A molecular modeling study on an RNase H1 enzyme from the hyperthermophile *Sulfolobus tokodaii* suggests that the extension of the C-terminus in the enzyme plays the sole role of stabilization by anchoring a range of disulfide bridges through the protein core.^4^ Thermally stabilizing C-terminal tails have also been discovered in the *Thermus* genus, where they may act as “clamps”, by a network of hydrophobic interactions on the surface of the cytochrome c_552_ protein.^5^ A range of 1,4-α-glucan-branching enzymes (GBEs) studied from the bacteria *Geobacillus thermoglucosidans* (optimal growth ∼60 °C) revealed a unique 26-amino acid extension of the C-terminus which is required for structural stability.^6^ A recent study of thermophilic DNA helicases show increased target binding due to an N- and C-termini extension, in comparison with nonthermophilic counterparts.^7^ C-Termini extensions have also been identified in Baker’s yeast (*Saccharomyces cerevisiae*), with a role in mitigating aggregation and deactivation of the structure.^8^ In particular, C-terminal tails with a helical conformation have been demonstrated as oligomeric stabilizers by increasing interactions at the surface of the molecule.^9^ α-Helical C-termini have also been propositioned to increase the rigidity of thermophilic proteases.^10^ Other studies have also probed into the stabilization phenomenon of an extended C-terminal tail in mesothermic proteins, uncovering its role in catalytic control and substrate specificity.^8,11−13^ For example, a fungal xylanase was found to possess a C-terminal extension, which decreased its catalytic efficiency by approximately 2.4 times.^13^ The catalytic activity of human ubiquitin-specific protease (USP) enzyme (USP7) has been shown to be tightly controlled by a 19-amino acid C-terminal extension, blocking excessive substrate binding.^14^

**Figure 1:**
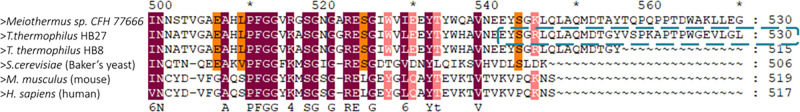
Sequence alignment of the C-termini of thermophilic and mesophilic aldehyde dehydrogenases. Alignment of sequences was performed using Clustal Omega and displayed using GeneDoc software. Purple areas represent regions of highly conserved homology. The blue dashed box displays the characteristic 30 amino acid tail of ALDH*Tt*.

An ALDH enzyme originating from a *Thermus thermophilus* bacterium (ALDH*Tt,* UniProtKB Q72KD3), which possesses a structurally unique C-terminal extension, has recently been discovered, during crystallization of a *caa*_*3*_-cytochrome c oxidase.^15^ The feature was deemed unique as it resembled the C-terminus extensions of dimeric ALDHs; however, its position over the catalytic tunnel linked it with the opposing monomer, forming a D2 or “222” dihedral point group symmetry. Additionally, the closely related *T. thermophilus* HB8 aldehyde dehydrogenase protein possesses a significantly shorter tail, with a full peptide length of 515 amino acids as opposed to 530 for ALDH*Tt*, yet retains its thermophilic properties. Alignment of the C-terminal tails of various ALDH proteins can be found in Figure 1. The C-terminal extension of ALDH*Tt* was recognized for its role in the protein’s stability by the creation of two mutants, ALDH*Tt*-515 and ALDH*Tt*-511 (PDB entry: 6FKU and 6FKV, respectively). The sequence of ALDH*Tt*-511 was deposited as ALDH*Tt*-508; however, further examination of the sequence by MALDI-TOF in this manuscript has revealed three additional amino acids, AQM, not truncated from the C-terminus. The FASTA sequence on PDB was wrongly deposited during this previous study, and we present the correct sequence for ALDH*Tt*-508 in Figure S1.

**Figure 2:**
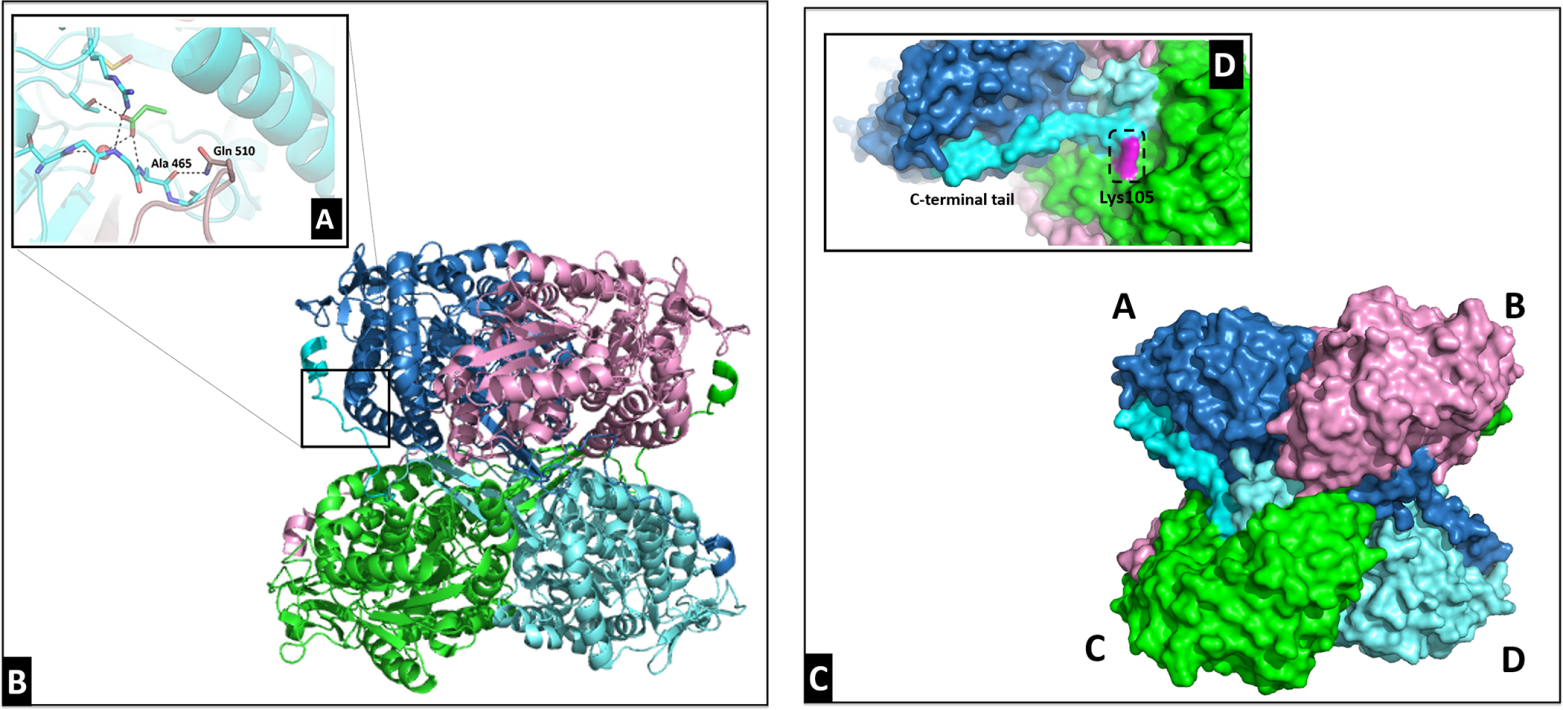
Relationship between the substrate access tunnel and C-terminal tail in ALDH*Tt*-native (A) and the ALDH*Tt*-native tetrameric structure as a ribbon model (B). All four monomers of the structure are labeled in the surface model of ALDH*Tt*-native in (C). The monomers are labeled as follows; A, blue; B, pink; C, green; and D, cyan. (D) Repulsion forces between Lys105 (shown in magenta) and the “hook” of the tail, causing the tail to extend toward the N-terminus of the relative monomer. Panel (A) was adapted from ref (15). Available under a CC-BY 4.0 license. Copyright 2018 Kevin Hayes et al.

The C-terminal tail of ALDH*Tt* consists of 30 amino acids (see Figure 1), encompassing a three-turn helix, made up of the last six residues in the sequence. Prolines residing at positions P518, P521, and P523 prevent the formation of secondary structure across the substrate access tunnel over which the extension is positioned.^16^ In Figure 2D, the relation between the substrate access tunnel and the unique extension of the tail is depicted, which is governed by the side chain of lysine at position 105 of the opposing monomer. The C-terminal tail in related ALDH proteins may be divided into three loops, or turns, termed the “notch”, the “crook” and the “hook”, respectively. Most dimeric ALDHs C-terminal tails end at the “notch”, which lays between the residues Ser503 and Gly504 in ALDH*Tt*. The C-terminal tail in ALDHTt however continues toward the N-terminus of the corresponding symmetrical monomer. Interestingly, the C-termini of aldehyde dehydrogenases produced by other closely related thermophilic bacterial strains are conserved until residue 515 (see Figure 1). Subsequent amino acids do not show any level of homology among the species. The “crook” is positioned directly over the substrate tunnel and governed by the interaction of Leu508 and Asp512. The residue Gln510 forms a hydrogen bond with an alanine, Ala465, located in the substrate anchoring region within the tunnel (Figure 2A). Through this strong, noncovalent interaction, the C-terminal tail of the ALDH*Tt* plays an important role in enzyme activity, unlike most other ALDH proteins where the access site is open, or the tail is repositioned during catalysis.^17^

The ALDH superfamily of enzymes have only recently been recognized for their application in biocatalysis, having the potential to replace chemical methods in the conversion of aldehydes to carboxylic acids, particularly due to their safety, ease of use, and minimal environmental impact.^18^ Exploration of the structure and function of ALDHs is vital to uncovering their potential applications in this area. Additionally, the presence of C-terminal tails has interesting implications for the structural and thermal stability of proteins and warrants further investigation. In this paper, a mutant of ALDH*Tt*-native is created with a fully open substrate tunnel by removal of the key 22 amino acids and 3-turn helix of the C-terminal extension, termed ALDH*Tt*-508. The effect on function and stability of the truncation is explored by screening of pH and temperature profiles, substrate specificity, size exclusion high-performance liquid chromatography (SE-HPLC), and dynamic light scattering (DLS). The data presented in this manuscript suggests that the C-terminus not only has a vital role in stabilization of the tetrameric structure but also plays a role in activity control. The characterizations described herein aid in understanding the effect of the C-terminus extension in bacterial thermophiles and possibly offer a new avenue to explore for protein stabilization.

## Materials and Methods

### Materials

LB and S.O.C media was obtained from Sigma-Aldrich. Ampicillin sodium salt, PageRuler prestained protein ladder, 10 to 180 kDa, imidazole (99%), ethylenediaminetetraacetic (EDTA), 1-step TMB blotting solution, and Porablot nitrocellulose membrane (0.45 μm) were obtained from ThermoFisher Scientific. Trizma base, potassium chloride, sodium chloride, magnesium chloride, magnesium sulfate, glucose, glycerol, calcium chloride, deoxyribonuclease 1 from bovine pancreas, nickle sulfate, hexanal (>98%), and nicotinamide adenine dinucleotide sodium salt were purchased from Sigma-Aldrich.

### Methods

#### Transformation, Expression, and Purification of ALDHTt-508

PET22b(+) plasmids containing the sequence gene for ALDH*Tt-*native, 530 amino acids, and ALDH*Tt*-508 were constructed, as previously described elsewhere.^15^ The plasmids were stored at −20 °C. *Escherichia coli* (BL21)DE3 cells were used for the recombinant production of both proteins. The construct containing the gene encoding for ALDH*Tt*-native and ALDH*Tt*-508 with a shortened C-terminal tail was transformed into chemically competent *E. coli* BL21 (DE3) cells using heat shock and incubation in S.O.C (super optimal broth with catabolite repression) media.

After an overnight culture in LB media, the cells were incubated in ZY autoinduction media, as previously described elsewhere.^19^ The culture was grown for 48 h at 25 °C under constant shaking at 200 rpm. The cells were collected by centrifugation at 6000 × *g* for 15 min in a 4 °C precooled rotor centrifuge. The cell pellet was resuspended in a Lysis buffer (Tris 20 mM, 2-mercaptoethanol 5 mM, imidazole 10 mM, NaCl 500 mM), 2 mg/mL DNase, 25 mg/mL lysozyme, and 1 M MgCl_2_). The solution was mixed gently on ice using a pipet and transferred to a −80 °C freezer for a minimum of 12 h.

Purification of ALDH*Tt*-508 was conducted by nickel affinity chromatography using a chelating Sepharose fast flow resin (Cytiva Life Sciences) charged with Ni^2+^ ions. Intracellular proteins were separated from lysate by centrifugation, heat-shocked at 65 °C for 5 min to remove unstable intracellular contaminants, and filtered using a 0.45 μm nylon filter. The protein was eluted from affinity chromatography by FPLC (ÄKTAprime plus, Cytiva Life Sciences) using an imidazole gradient. Absorbance was monitored at 280 nm. The protein was dialyzed overnight using cellulose dialysis tubing, MWCO 8000 Da (Fisher Scientific), to remove imidazole from the storage buffer. The protein was snap-frozen and stored at −80 °C for further analysis. Size exclusion chromatography was conducted to evaluate the effect of lysate heat-shock on the protein using a pre-equilibrated HiLoad 16/60 Superdex 200 prep grade column (GE Healthcare). The mobile phase used during equilibration and elution consisted of 50 mM Tris-HCl pH 7.5, 5 mM β-mercaptoethanol, and 150 mM NaCl.

ALDH*Tt*-native was prepared as described elsewhere.^15^ The purity and presence of expressed protein was confirmed by 12% SDS-PAGE and Western blot, which utilized conjugation of Anti 6× His-peroxidase antibody to one-step TMB substrate solution (ThermoFisher Scientific). Quantitative purity was confirmed by SE-HPLC, by injecting 50 μL of a 0.5 mg/mL sample onto a SE-HPLC column (AdvanceBio SEC 300 Å, 2.7 μm, 8 × 300 mm, Agilent Technologies) at a flow rate of 1 mL/min. The purity of the samples was calculated by calculating the percentage of the native protein peak area to the total peak area.

#### MALDI-TOF

The samples were buffer exchanged into 0.1% trifluoroacetic acid (TFA) and concentrated to 3 mg/mL. Approximately 7.6 mg of 2,5-dihydroxyacetophenone (2,5DHAP) was dissolved in a 500 μL solution of 75:25 v/v ethanol, 18 mg/mL diammonium hydrogen citrate in water to make up the matrix solution. A 1 μL sample solution was mixed with 1 μL of 2% TFA solution and 1 μL of matrix solution. A 1 μL drop was deposited on a Bruker ground steel target plate and allowed to dry at room temperature. Intact mass spectra were obtained in a Bruker UltrafleXtreme MALDI TOF/TOF device equipped with a SmartBeam2 laser in linear positive mode with an acceleration gain of 20 kV, 500 ns ion extraction delay, and 2750 V detector gain. Data was acquired and processed using the Compass 1.4 software suit from Bruker. Peak masses were assigned using the Centroid algorithm in FlexAnalysis, and data were externally calibrated using the Protein Standard II from Bruker.

#### pH and Temperature Activity Parameter Screening

The pH and temperature stability of ALDH*Tt*-508 was tested using a range of temperatures and pH, previously described for ALDH*Tt* for the evaluation of the impact of the mutation on the pH and temperature activity profile of the protein.^18^ A temperature range of 20–50 °C and pH range of 2–10 were analyzed for NAD+ dependent activity, using the substrate hexanal. Temperatures above 50 °C were not analyzed due to the volatility of hexanal. pH ranges of 2–10 were achieved using a range of 10 mM citrate buffers (2–5), 10 mM potassium phosphate buffers (6–8), and 10 mM Tris-HCl buffers (9–10).

#### NAD+ Coupled Enzymatic Assay

The enzyme assays for both ALDH*Tt*-native and ALDH*Tt*-508 were conducted using 60 nM ALDH, 0.4 mM NAD+ (Nicotinamide adenine dinucleotide sodium salt, Sigma-Aldrich), and 1 mM of the substrate hexanal (Sigma-Aldrich) in a volume of 1 mL. All solutions were made in 10 mM potassium phosphate buffer (pH 8.0), and the rise in absorbance was measured every 5 s for 120 s. All enzyme assays were conducted in triplicate. The enzyme activity was calculated by the change in NADH concentration in the solution per minute and expressed in 1 U/mg (specific enzyme units), where 1 U = 1 μmol of NADH/min. All enzymatic assays were performed using an Evolution 201 Series UV–vis spectrophotometer (ThermoFisher). Kinetic parameters were evaluated using GraphPad Prism 8.0.1.

#### Substrate Screening

A range of aldehydes were screened to observe the effect of the loss of the C-terminal extension on ALDH*Tt* substrate specificity and affinity. The experiments were conducted at room temperature (25 °C) and at the optimum operating temperature of the enzyme (50 °C). Approximately 2 mM of the following aromatic and aliphatic aldehydes were used in the experiments: cyclo-hexanecarboxaldehyde, furfural, acetaldehyde, benzaldehyde, *trans*-cinnamaldehyde, *o*-tolualdehyde, *p*-tolualdehyde, propanal, and citral.

#### Oligomeric Stability Testing

SE-HPLC was conducted on heat-treated ALDH*Tt*-native and ALDH*Tt*-508 samples to probe the oligomeric stability of the proteins. All samples were injected at room temperature onto a SE-HPLC column (AdvanceBio SEC 300 Å, 2.7 μm, 8 × 300 mm, Agilent Technologies) at a flow rate of 1 mL/min using an Agilent 1260 Infinity Series HPLC system (Agilent Technologies). The signals were collected by UV absorption at 220 and 280 nm.

The samples were adjusted to 0.5 mg/mL in 10 mM potassium phosphate buffer and incubated on a block heater at 65 °C for 5, 10, 15, and 20 min. The samples were then centrifuged at 10000 × *g* for 10 min to remove large particles. The column was calibrated with the following mixture: Thyroglobulin bovine MW ∼ 670 kDa, γ-globulins from bovine blood with MW ≈ 150 kDa, ovalbumin with MW ≈ 44.3 kDa, ribonuclease A type I-A with MW ≈ 13.7 kDa, and *p*-aminobenzoic acid (pABA) with MW ≈ 137 Da. Approximately 50 μL of each sample was injected into the column, which was pre-equilibrated with 10 mM potassium phosphate buffer. The molecular masses of the proteins and their oligomeric states were determined from the calibration plot of the standards.

#### Dynamic Light Scattering (DLS)

The steady-state hydrodynamic diameter (*d*_H_) of proteins ALDH*Tt*-native and ALDH*Tt*-508 was measured using dynamic light scattering (DLS) on a Zetasizer Nano-ZSP (Malvern Instruments, UK) at 25 °C in three independent experiments. The samples were prepared by dilution or buffer exchange into 10 mM potassium phosphate buffer at a concentration of 800 μg/mL and filter-sterilized by a 0.22 μm PTFE filter to remove large aggregates. The intensity of scattered light was measured at 173° with an avalanche photodiode. The instrument was fitted with a 633 nm He–Ne laser. The hydrodynamic diameter was calculated from the Stokes–Einstein equation:^20^
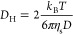
1where *D*_H_ is the hydrodynamic radius, *T* is absolute temperature (varied), η_s_ is the solvent viscosity (10 mM potassium phosphate buffer was used as solvent), and *k*_B_ is the Boltzmann constant. The measurements were taken at a scattering angle of 90° to reduce scattering from dust in the solution. The temperature of aggregation (*T*_agg_) was determined using multiparameter analysis (monitoring both size and intensity of scattered light as a function of temperature). The temperature was increased from 30 to 70 °C with a 0.5 °C step increment and a 60 s equilibration time of the sample at each temperature step. The *T*_agg_ was determined by fitting the experimental data to a plateau followed by a one-phase association model (eq 2) using Prism software (version 10.0.1).
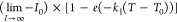
2where *T*_0_ is the time at which an increase in intensity is observed (*T*_agg_),  is the maximum intensity reached during the experiment, and *k*_I_ is the kinetic rate constant of aggregation.

All measurements were conducted in a minimum of two independent experiments.

Thermal aggregation was measured by measuring the scattering intensity and *D*_H_ of the sample as a function of time at the respective *T*_agg_ of each protein, 65 and 80 °C. The aggregation kinetics were fit to an exponential growth curve using Prism software (ver 10.0.1).

#### Circular Dichroism (CD)

Untreated secondary structure spectra of ALDH*Tt-*native and ALDH*Tt*-508 were analyzed using a Chirascan Plus CD spectrophotometer (Applied PhotoPhysics Ltd., UK). The spectra were recorded in the far-UV wavelength range 180–250 nm. The path length was 1 cm with a wavelength step of 1 nm. Wavelength scans were obtained in triplicate of protein solutions of 0.005 mg/mL in 10 mM potassium phosphate buffer, pH 8.0, the spectra of which were also used as a blank. A quartz cuvette was used for the analysis. All spectra were reported in CD units (mdeg). The spectra were averaged, background corrected using the blank, and allowed 6-point Savitsky–Golay smoothing using the accompanying software Pro-Data Chirascan. Secondary structure composition of both proteins was estimated using the K2D2 predictor tool available online at dichroweb.cryst.bbk.ac.uk.^21^

## Results

### Recombinant Production and Purification of the Kinetically Active Mutant ALDH*Tt*-508 and Assessment of Lysate Heat-Treatment

**Figure 3:**
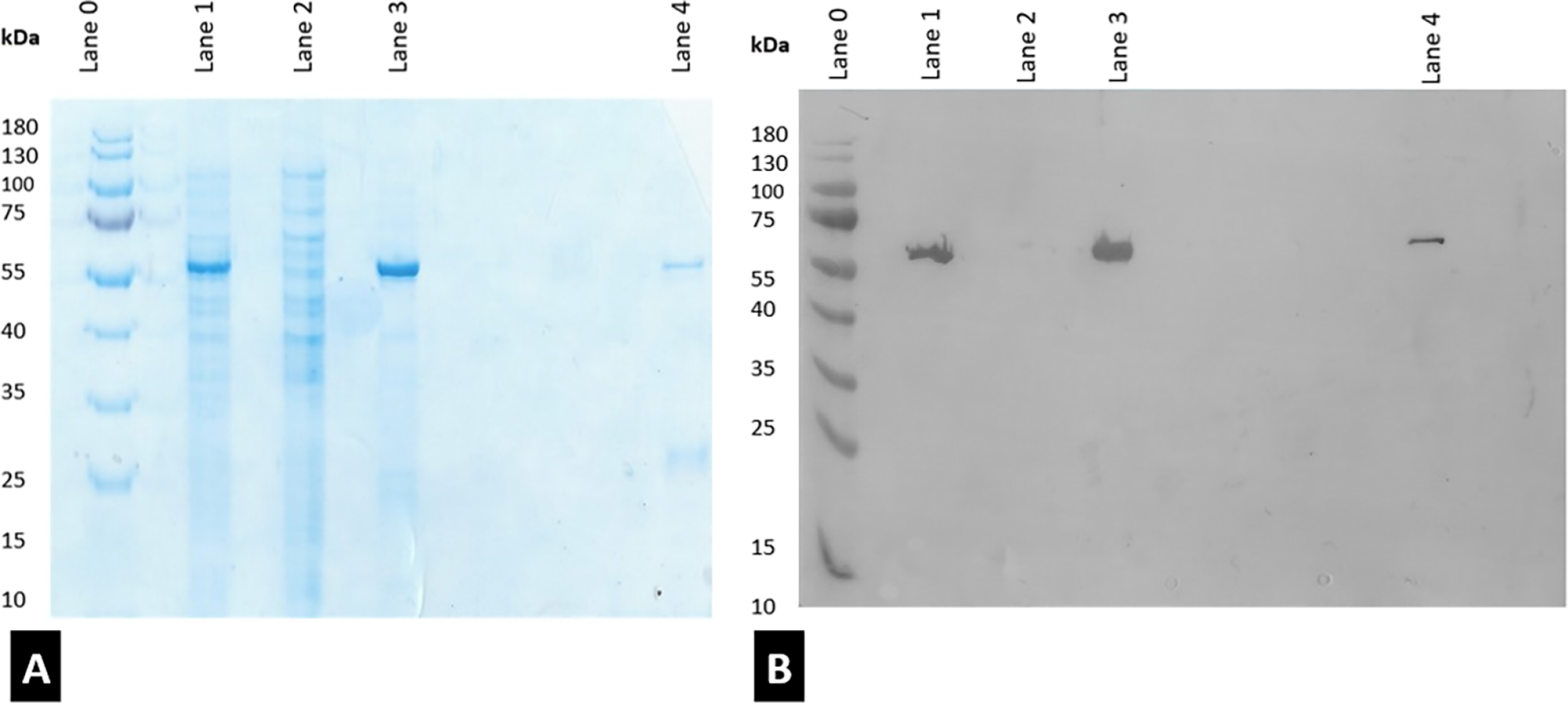
SDS Page (A) and Western blot gels (B) from the production of ALDH*Tt*-508. (Lane 0) molecular weight marker (PageRuler Prestained Protein ladder, ThermoFisher), (lane 1) cell lysate, (lane 2) nickel affinity eluted waste, (lane 3) elution at 200 mM Imidazole, and (lane 4) elution at 500 mM imidazole.

The overexpression of ALDH*Tt*-508 in *E. coli* cytosol was confirmed by a strong band present in the lysate soluble fraction made up of *E. coli* intracellular proteins at approximately 57–58 kDa via SDS-PAGE (Figure 3A, lane 1) and confirmed to contain the His-tagged mutant by Western blot (Figure 3B, lane 1). The purity of samples after Ni-affinity was confirmed to be 89.9 ± 0.2%, as calculated by ratio of peak area of protein to contaminants by SE-HPLC (see Table 1 below) and qualitatively by 12% SDS-PAGE (Figure 3A, lane 3). The protein was seen to fully bind to the resin as little to no protein is visualized in the nickel-affinity flow-through (Figure 3A, lane 2). The protein of interest eluted similarly to native ALDH*Tt*, at 200 mM imidazole (Figures S2 and S3). Most contaminating *E. coli* proteins are seen eluted between 0 and 50 mM imidazole, as per Figure S2. Samples subjected to SEC confirm the purity and structural integrity of the protein after affinity chromatography by a singular peak at an 80 mL elution volume (Figure S3B). The structural integrity of the tetramer was retained under varied levels of lysate heat shock (0–15 min), as confirmed by a singular peak at 80 mL elution volume during gel filtration (Figure S7); however, the yield was majorly affected by the lack or excess of heat treatment of the lysate (Table 1).

**Table 1:** Purification Profile of ALDH*Tt*-508

purification step	lysate heat treatment at 65 °C (min)	protein	yield (mg/L)	purity (%)	specific enzyme activity (U/mg)
affinity	0	ALDH*Tt*-508	25.8 ± 2.6	82.7 ± 17.2	0.750 ± 0.184
	5	ALDH*Tt*-508	22.6 ± 8.6	89.9 ± 0.2	0.564 ± 0.120
	15	ALDH*Tt*-508	19.3 ± 5.6	87.1 ± 0.5	0.306 ± 0.021
gel filtration	0	ALDH*Tt*-508	8.5 ± 3.5	100.0 ± 0.0^a^	0.545 ± 0.037
	5	ALDH*Tt*-508	24.5 ± 7.6	96.6 ± 0.4	0.953 ± 0.035
	15	ALDH*Tt*-508	14.4 ± 4.0	6.4 ± 1.5^b^	0.453 ± 0.041
affinity	15	ALDH*Tt*-native	66.5 ± 10.5	97.5 ± 0.1	0.432 ± 0.035
gel filtration	15	ALDH*Tt*-native	57.2 ± 10.2	98.4 ± 0.0	0.636 ± 0.039

a As detectable by the described SE-HPLC method at 280 nm.

b Represents percentage of fully folded protein corresponding to the tetrameric peak of ALDH*Tt*-508.

Table 1 shows the details of the effect of various purification approaches used. Each 1 L of ZY-auto induction culture purified by a single affinity chromatography step yielded on average 22.6 ± 8.6 mg/L, when the cell lysate was treated to 5 min of heat shock. The yields were approximately 45% lower than those obtained from the production of ALDH*Tt-*native (66.5 ± 10.5 mg/L). The protein, which was treated to only 5 min of heat treatment, was relatively homogeneous after Ni-affinity, achieving 89.9% purity. Treatment of the lysate to a 15 min heat shock step, as described elsewhere for production of native ALDH*Tt*,^15,18^ drastically lowers the specific activity of ALDH*Tt*-508 after purification by Ni affinity and gel filtration. This technique is common for purification of thermozymes expressed in mesophilic hosts.^22^ The truncated enzyme displays a specific activity of 0.453 ± 0.041 U/mg when purified to homogeneity by gel filtration after a 15 min heat shock step. The enzyme activity of ALDH*Tt*-508 increases to 0.953 ± 0.035 U/mg when this step is reduced to 5 min. Complete omittance of this step does not improve the retention of enzymatic activity, with activity reaching 0.545 ± 0.037 U/mg. Furthermore, treatment of ALDH*Tt*-508 to a 15 min heat shock step results in a deterioration of the protein, resulting in two distinct peaks detectable in SE-HPLC (Figure S8). The major peak present in heat-treated lysate samples was the native tetramer (27.02% of total peak area); however, the second biggest peak (6.65% of total peak area) corresponded to the molecular weight of an ALDH*Tt*-508 hexamer (MW ≈ 380 kDa). Other peaks corresponded to trimeric (∼190 kDa) and dimeric (118 kDa) species.

The catalytic activity is improved for ALDH*Tt*-508 when the heat shock purification step is optimized. Previously, the production of ALDH*Tt*-native has been optimized to include a 15 min heat shock step, with reduction of this purification step resulting in a 40% loss of yield.^15,18^ When both proteins are treated to a 15 min heat shock step, the activity of ALDH*Tt*-508 drops to ∼70% of that achievable by ALDH*Tt*-native, which prompted optimization of this step in production of ALDH*Tt*-508. Reduction of the lysate heat treatment time of ALDH*Tt*-508 improved yields by 58% and improved the enzymatic activity by ∼50% (see Table 1). Indeed, affinity purification of ALDH*Tt*-native under 5 min lysate heat treatment resulted in a specific activity of 0.542 ± 0.011 U/mg, slightly lower than that of ALDH*Tt*-508 treated to the same purification steps (0.564 ± 0.120, see Table 1). The purity of ALDH*Tt*-508 is marginally improved by a second gel filtration step, increasing from 82–90 to 96–100% (Table 1). There is no significant difference in purity of the protein when treated to 0 or 5 min of heat treatment, and therefore, the step does not serve any beneficial function in the process. Retention of the 15 min heat treatment had a profound impact on purity, due to the presence of nonspecific oligomer states (aggregates) and cleaved protein states. The cleavage of ALDH*Tt*-508 at increased temperatures is not related to the 6xHis-tag, as suspected, as no irregular elutions were noticed during nickel affinity purification.

### Truncation of the C-Terminus Broadens the Substrate Specificity of ALDH*Tt*

The presence and molecular mass of the protein was confirmed by MALDI-TOF (Figure S4). The molecular mass of the 508 amino acid protein was 57055.97 Da, as measured by MALDI-TOF, comparable to the theoretical molecular mass returned by the Expasy Molecular Weight Analysis Tool, 57079.02 Da (https://web.expasy.org, accessed 26th of April, 2023). This confirmed the successful expression of the mutant protein.

**Figure 4:**
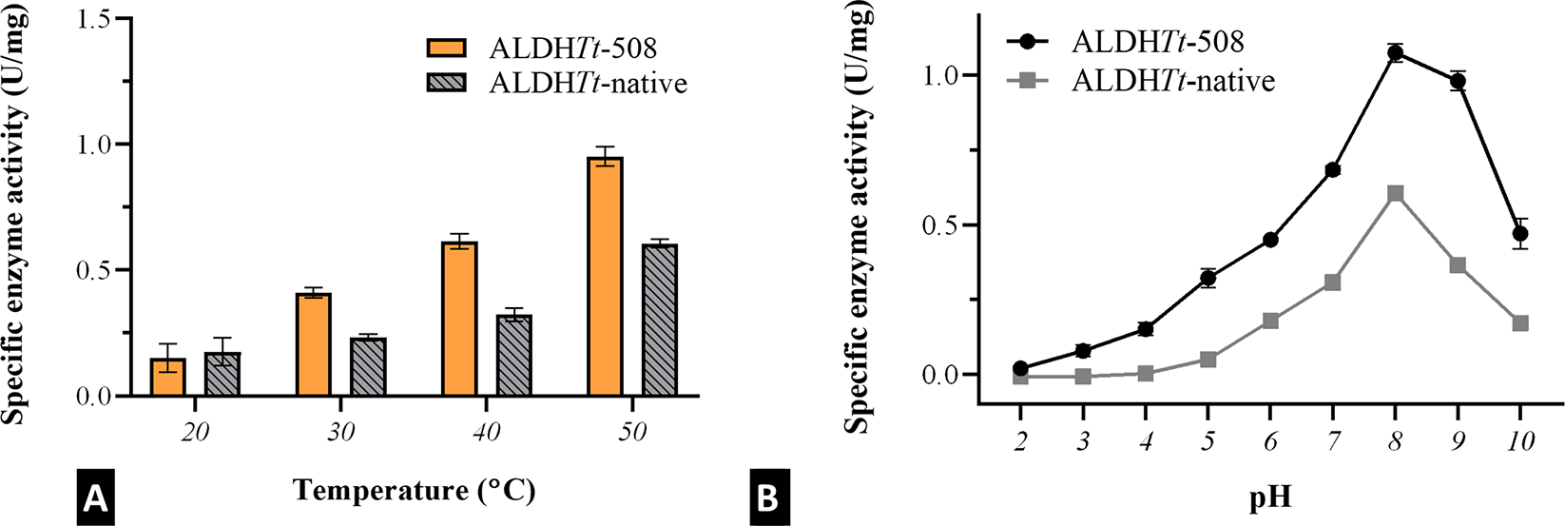
Temperature (A) and pH (B) profiles of ALDH*Tt*-508 using an NAD+ coupled assay and hexanal as the substrate.

Due to the position of the C-terminal tail over the substrate access tunnel in the oligomeric state and the apparent impact on the catalytic rate of the protein, a detailed pH and temperature profile was conducted. The pI of ALDH*Tt*-508 remained largely unchanged upon the mutation (pI_ALDHTt-508_ = 6.53, pI_ALDHTt-native_ = 6.40), and therefore, no major change was observed for the optimum working pH of the protein (Figure 4B). The temperature profile of ALDH*Tt*-508 is largely similar to that of ALDH*Tt*-native, with a gradual increase to 0.952 ± 0.032 U/mg specific enzyme activity at 50 °C, showcasing the mutant’s suitability to high-temperature catalytic applications (Figure 4A). The mutant protein displayed higher rates of activity at temperatures of 30, 40, and 50 °C, albeit the truncation did not affect activity at lower temperature. Similarly, the pH profile resembles the pH profile of ALDH*Tt*-native, with an optimum operating pH of 8.0 (1.074 ± 0.031 U/mg). ALDH*Tt*-508 was active at pH 3–10, while ALDH*Tt*-native showed activity only in the pH range of 5–10. The mutant protein is more active than ALDH*Tt*-native at all pHs tested.^18^

Optimum catalytic conditions for the enzyme, using hexanal as a substrate, were found to be 50 °C at pH 8.0. As native ALDH*Tt* follows Michaelis–Menten kinetics, values for *K*_cat,_*V*_max_, and *K*_m_ were calculated for the ALDH*Tt*-508 protein at the experimentally derived optimum pH and temperature and at 25 °C (room temperature). Results clearly indicate the thermophilic application of ALDH*Tt*-508, with a 3.6-fold increase in the rate of reaction (*V*_max_) from 25 to 50 °C and a proportional increase in the amount of NAD+ reduced per second (*K*_cat_) of 3.8-fold (Table 2, Figure S5). Additionally, the enzyme reaches maximum velocity at much lower concentrations of substrate at 50 °C, indicated by the decrease in the halfway point of the reaction, *K*_m_, from 1.0078 ± 0.6021 mM at 25 °C to 0.1040 ± 0.0093 mM at 50 °C. The kinetic activity of ALDH*Tt*-508 using the cofactor NADP+ was also calculated at 50 °C using hexanal. The enzyme was significantly less active using the larger cofactor, with a specific activity of 0.1915 ± 0.0341 U/mg (Figure S6, Table 2). When compared with an ALDH*Tt*-native control, ALDH*Tt*-508 showed more robust kinetic parameters using NAD+ as a cofactor at both mesophilic and thermophilic temperatures. A minor increase in the activity of the truncated enzyme was also observed when the larger cofactor, NADP+, was used in the assay.

**Table 2:** Calculated Kinetic Parameters for the Mutant Enzyme ALDH*Tt*-508 and the Native Enzyme ALDH*Tt*-native Using Hexanal as the Substrate and NAD+ or NADP+ as the Cofactor^a^

temperature	protein	cofactor	*K*_m_ (mM)	*V*_max_ (mM/min)	*K*_cat_ (s^–1^)	U/mg (μmol/mg/min)
25 °C	ALDH*Tt*-508	NAD+	1.0078 ± 0.6021	0.0008 ± 0.0003	13.54 ± 4.12	0.1724 ± 0.0275
25 °C	ALDH*Tt*-native	NAD+	0.1456 ± 0.0212	0.0005 ± 0.0000	9.20 ± 0.30	0.1402 ± 0.0263
50 °C	ALDH*Tt*-508	NAD+	0.1040 ± 0.0093	0.0029 ± 0.0001	49.70 ± 5.05	0.8138 ± 0.0085
50 °C	ALDH*Tt*-native	NAD+	1.9835 ± 0.8591	0.0028 ± 0.0009	46.84 ± 14.68	0.6060 ± 0.0162
50 °C	ALDH*Tt*-508	NADP+	0.4871 ± 0.2270	0.0009 ± 0.0001	12.53 ± 1.61	0.1915 ± 0.0341
50 °C	ALDH*Tt*-native	NADP+	0.0917 ± 0.0020	0.0006 ± 0.0000	10.26 ± 0.29	0.1453 ± 0.0022

a The final volume of the assay was 1 mL.

**Figure 5:**
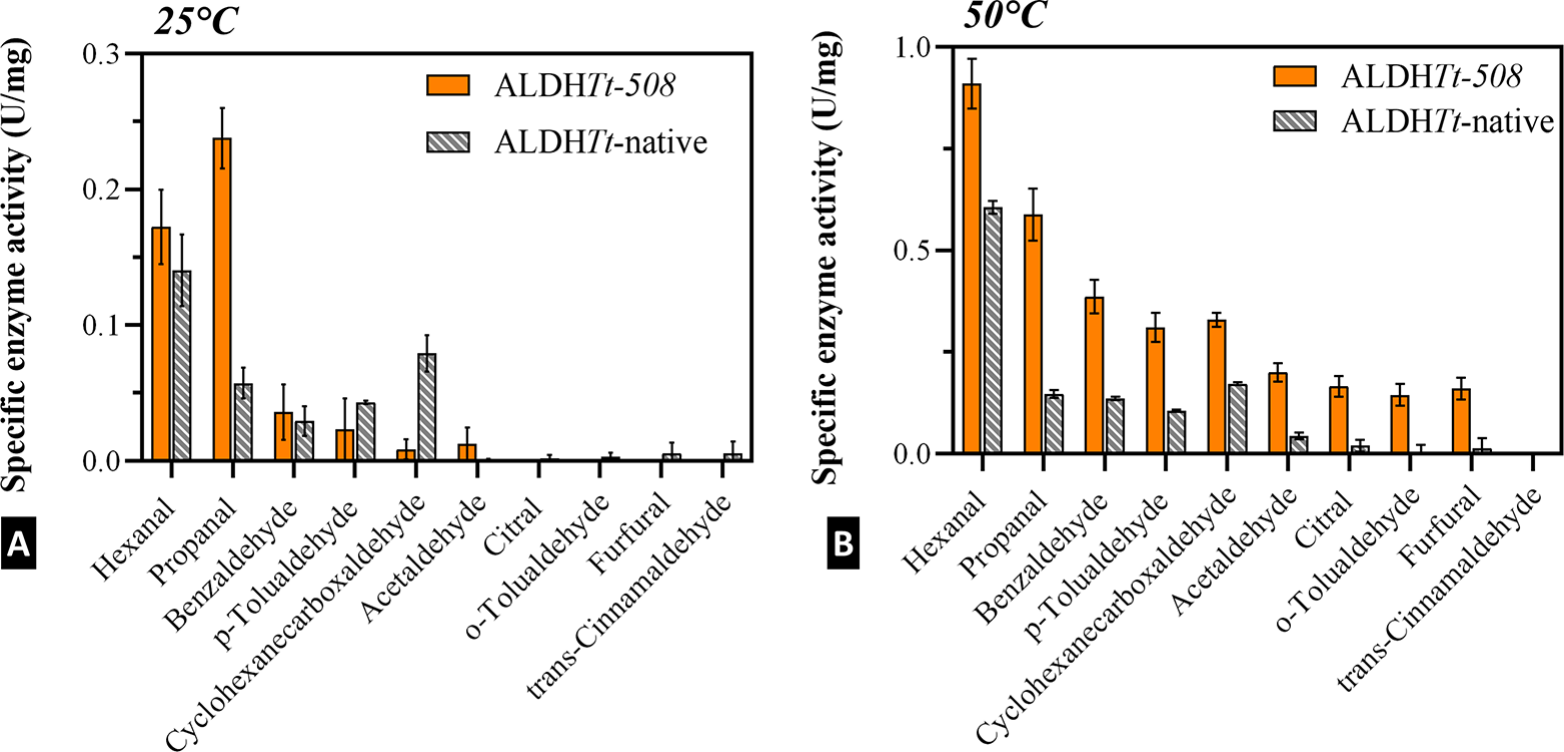
Screening of ALDH*Tt*-508 aldehyde specificity at 25 °C (A) and 50 °C (B). The enzyme has a broad substrate specificity toward a range of aldehydes, particularly at higher temperatures.

ALDH*Tt-*native has recently been reported as having a wide substrate specificity toward both aromatic and aliphatic aldehydes.^18^ To observe if the truncation of the C-terminus influenced substrate specificity, a range of aliphatic and aromatic aldehydes were screened at 25 and 50 °C, pH 8.0 (Figure 5). Figure 5B shows that the truncated mutant, ALDH*Tt*-508, is capable of oxidizing both aliphatic (hexanal, propanal, acetaldehyde, and citral), aromatic (benzaldehyde, *p*-tolualdehyde, *o*-tolualdehyde, and furfural), and cyclic (cylohexaneboroaldehyde) compounds at a thermophilic temperature of 50 °C. The mutation at the C-terminus alters the specificity and activity of the enzyme toward the tested aldehydes. Removal of the C-terminus causes a total loss of enzymatic activity toward the aromatic compound *trans*-cinnamaldehyde at mesophilic temperatures (Figure 5). The enzyme retains a similar, minimal, affinity toward benzaldehyde at both 50 and 25 °C; however, it gains a substantial affinity toward propanal, and to a lesser extent, citral and furfural. At 50 °C, ALDH*Tt*-508 shows a greater activity toward all of the aldehyde substrates tested. ALDH*Tt*-508 also possesses a broader substrate scope than ALDH*Tt* at 50 °C operating temperature in pH 8.0, capable of oxidizing *o*-tolualdehyde, citral, and furfural. The increase in specific enzymatic activity for the cyclic compound, cyclohexanecarboxaldehyde, is 3-fold, while affinity toward the aliphatic propanal increases by 6-fold. Unlike ALDH*Tt*-native, ALDH*Tt*-508 displays a higher activity toward propanal (0.238 ± 0.022 U/mg) than hexanal (0.172 ± 0.027 U/mg) at room temperature. The truncated enzyme is significantly more active toward the aliphatic aldehydes hexanal and propanal at room temperature than ALDH*Tt*-native. A plot of the relative activity of each protein toward each substrate tested, with respect to the model substrate, hexanal, can be found in the Supporting Information (Figure S7).

### Influence of the C-Terminal Tail on the Oligomeric and Thermal Stability of ALDH*Tt*

Oligomeric dissociation and aggregation during thermal stress to both proteins, with and without a C-terminal extension, was examined using SE-HPLC and DLS. Determination of the oligomeric forms present in purified solutions of ALDH*Tt*-native and ALDH*Tt*-508 showed relative instability of ALDH*Tt*-508 in solution, and a tendency toward monomeric (2.50%) and dimeric (1.71%) oligomeric states (see Table 3). There was also an increment of aggregates in solution for ALDH*Tt*-508, increasing from 1.15% for ALDH*Tt*-native to 5.92% for ALDH*Tt*-508 in 10 mM potassium phosphate buffer, pH 8.0. Incubation of the protein solutions at 65 °C led to a gradual loss of tetrameric structure for the native protein ALDH*Tt*-native, decreasing from 98.8 to 93% over a period of 20 min, with a fraction shifting into soluble aggregates. SE-HPLC of ALDH*Tt*-508 was not representative of the behavior of the protein in solution. For samples heated from 5 to 15 min at 65 °C, there is a decrease in the number of soluble aggregates in solution; however, extensive precipitation of the protein, which was filtered prior to injection, contradicted this result. The decrease in monomeric and dimeric states upon heating suggests that the structural fragments are involved in the formation of large insoluble aggregates. The hydrodynamic diameter, as determined by DLS, displayed values of 11.44 ± 0.24 nm for the native protein and 14.40 ± 2.37 nm for ALDH*Tt*-508, which is in accordance with the diameter reported for other globular proteins of similar molecular weight and with the predicted molecular weight using the Zetasizer Nano-ZSP instrument (264 and 212 kDa, respectively).^23^ The values of experimentally derived *D*_H_ reported are a weighted average obtained from the Zetasizer Nano-ZSP instrument, which creates a bias toward larger (aggregate) populations.^24^ The higher hydrodynamic diameter reported for ALDH*Tt*-508 is therefore likely to be caused by the higher presence of aggregates in the sample (5.9% as measured by SE-HPLC) which were not filtered (*D*_H_ < 0.22 μm). The size distribution by intensity of ALDH*Tt*-508 shows three main populations present, at 11.14 ± 0.43 nm, 176.62 ± 86.39 nm, and >2 μm, with peak 1 corresponding to ∼90% of the total distribution area (Figure S9). This indicates that the true *D*_H_ of ALDH*Tt*-508 corresponds to 11.14 ± 0.43 nm. Both the SE-HPLC and DLS results show a destabilization of the tetrameric structure of ALDH upon C-terminal removal and a tendency toward formation of monomeric and dimeric species in solution.

**Table 3:** Distribution of Oligomeric States and Steady-State Hydrodynamic Diameter (*D*_H_) of Native and Truncated Aldehyde Dehydrogenase (ALDH)^d^

		oligomeric states (%)				
sample		monomer	dimer	tetramer	other	*D*_H_^a^ (nm)
ALDH*Tt*-native, control		0	0	98.84 ± 0.28	1.15 ± 0.27	11.44 ± 0.24
incubation time at 65 °C (min)						
5		0	0	97.65 ± 0.02	2.35 ± 0.02	
10		0	0	94.89 ± 0.06	6.01 ± 0.47	
15		0	0	93.70 ± 0.04	6.31 ± 0.06	
20		0	0	93.15 ± 0.06	6.85 ± 0.06	
ALDH*Tt*-508, control		2.50 ± 0.03	1.71 ± 0.01	89.99 ± 0.29	5.92 ± 0.40	11.14 ± 0.43^c^
incubation time at 65 °C (min)						
5		0.87 ± 0.07	0	95.4 ± 2.07	3.56 ± 1.70	
10		0.79 ± 0.12	0	93.09 ± 0.08	6.11 ± 0.06	
15		0.55 ± 0.08	0	95.78 ± 0.31	3.88 ± 0.36	
20		0.71 ± 0.27	0	91.37 ± 11.31	7.65 ± 11.04^b^	

a The hydrodynamic diameter (*D*_H_) was determined by DLS.

b Larger soluble aggregates were visualized in only one experiment.

c The hydrodynamic diameter corresponds only to the tetrameric population of ALDH*Tt*-508.

d Oligomeric states were detected by SE-HPLC in triplicate. The control refers to untreated protein, injected directly after thawing.

**Figure 6:**
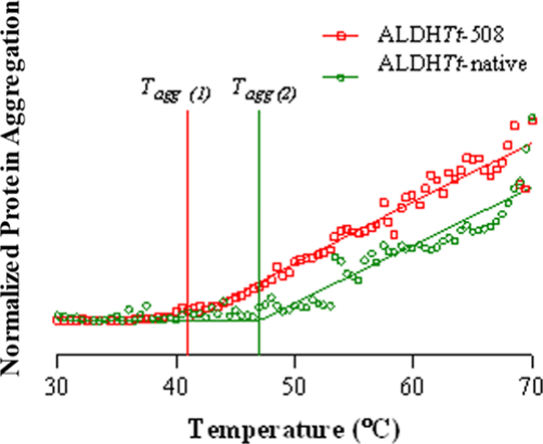
Effect of the loss of the C-terminal extension on the aggregation profiles of ALDH*Tt*-native and ALDH*Tt*-508 measured by dynamic light scattering (DLS). The intensity and hydrodynamic diameter data was collected using the Zetasizer Nano-ZSP DLS and normalized using Prism GraphPad (Ver 10.0.1) and fit to a plateau, followed by a one-phase association equation. The mean of three independent experiments is presented.

The stability of the oligomeric ALDH*Tt*-native and its mutant, ALDH*Tt*-508, was studied using dynamic light scattering (DLS). The aggregation profiles of both proteins as a function of the temperature are shown in Figure 6. There was a significant change in both the increasing hydrodynamic diameter (*D*_H_) and the scattering light intensity (kcps) as a function of temperature between the two proteins. The onset of aggregation for ALDH*Tt*-native was determined at 47 °C, 6 °C higher than that of ALDH*Tt-*508 (41 °C). Additionally, heating of ALDH*Tt*-508 to 70 °C resulted in an increase in the average *D*_H_ to >2 μm due to the formation of precipitates, while the *D*_H_ of the native protein increased to only 24 nm. This is reflected by the magnitude of scattering intensity measured by DLS (ALDH*Tt*-native^70 °C^ = 4,525 kcps, ALDHTt-508^70 °C^ = 31,487 kcps, data not shown).

**Figure 7:**
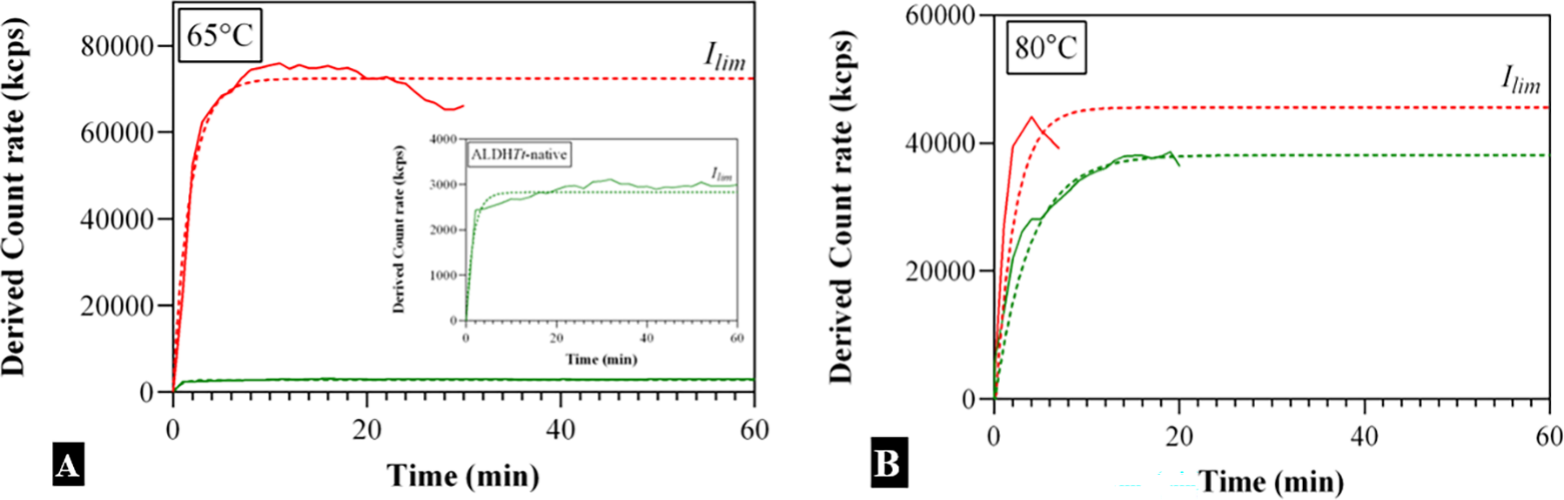
Aggregation kinetics of ALDH*Tt*-native (green) and ALDH*Tt*-508 (red) at temperatures of 65 (A) and 80 °C (B), as measured by dynamic light scattering (DLS). The intensity of diffraction is presented as a function of time. The samples were prepared at 800 μg/mL in 10 mM KPO_4_ buffer and filter sterilized. The experimental data points (solid lines) show the mean of at least two independent experiments. The result of fitting (eq 3) is shown by dotted lines. *I*_lim_ is defined as the final point of aggregation that can be detected by DLS.

Aggregation kinetics of both ALDH*Tt*-native and ALDH*Tt*-508 was measured by DLS at 65 and 80 °C (Table 4, Figure 7). The aggregation mechanisms of both proteins followed an exponential model of association at the initial stages of aggregation. The increase in intensity for both proteins reached a plateau, and therefore can be defined by the following equation:
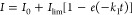
3where *I*_lim_ is the limiting intensity [*I*] at *t → ∞* and *k*_1_ is the first-order rate constant. Neither of the proteins show evidence of intermediate formation upon thermal stress, either in SE-HPLC or DLS as suggested previously for a structurally similar ALDH from *Pseudomonas aeruginosa* (PaBADH).^25^ This leads us to the conclusion that ALDH*Tt* follows a monomolecular aggregation mechanism with no creation of intermediate oligomeric species. At temperatures of 65 and 80 °C, the presence of the C-terminal tail in ALDH*Tt*-native slows the rate of aggregation (*k*_I_) by 3-fold and 2-fold, respectively. The presence of the C-terminal extension also prevents the formation of large insoluble aggregates at 65 °C.

**Table 4:** Thermal Aggregation Kinetics of Aldehyde Dehydrogenase (ALDH) Native (ALDH*Tt*-native) and without the Native C-Terminal Extension (ALDH*Tt*-508) at 65 and 80 °C

	65 °C		80 °C	
	*k*_1_ (min^–1^)	*I*_lim_ (kcps)	*k*_1_ (min^–1^)	*I*_lim_ (kcps)
ALDH*Tt*-native	1.43 ± 0.43	2808.0 ± 674.2	0.23 ± 0.16	40,756.6 ± 15,136.6
ALDH*Tt*-508	0.57 ± 0.07	72,402.5 ± 18,707.9	0.57 ± 0.07	66,136.6 ± 18,707.9

For ALDH*Tt*-508 at 65 and 80 °C, the ascending kinetics at the beginning of the experiment follow the same exponential model shown in eq 3, where the *I*_lim_ value denoted the point at which all protein was involved in aggregate formation; however, there is a gradual descent of the curve past the point of *I*_lim_, suggesting the formation and sedimentation of precipitates (*D*_H_ > 2000 nm), which are not detected using the DLS technique.^26,27^ At 80 °C, both proteins precipitate out of solution, with ALDH*Tt*-native precipitating at ∼21 min, compared to 6 min for ALDH*Tt*-508. ALDH*Tt*-native possessed a lower value of *I*_lim_ for both temperatures studied (2808.0 ± 674.2 kcps at 65° and 40,756.6 ± 15,136.6 kcps at 80°).

**Figure 8:**
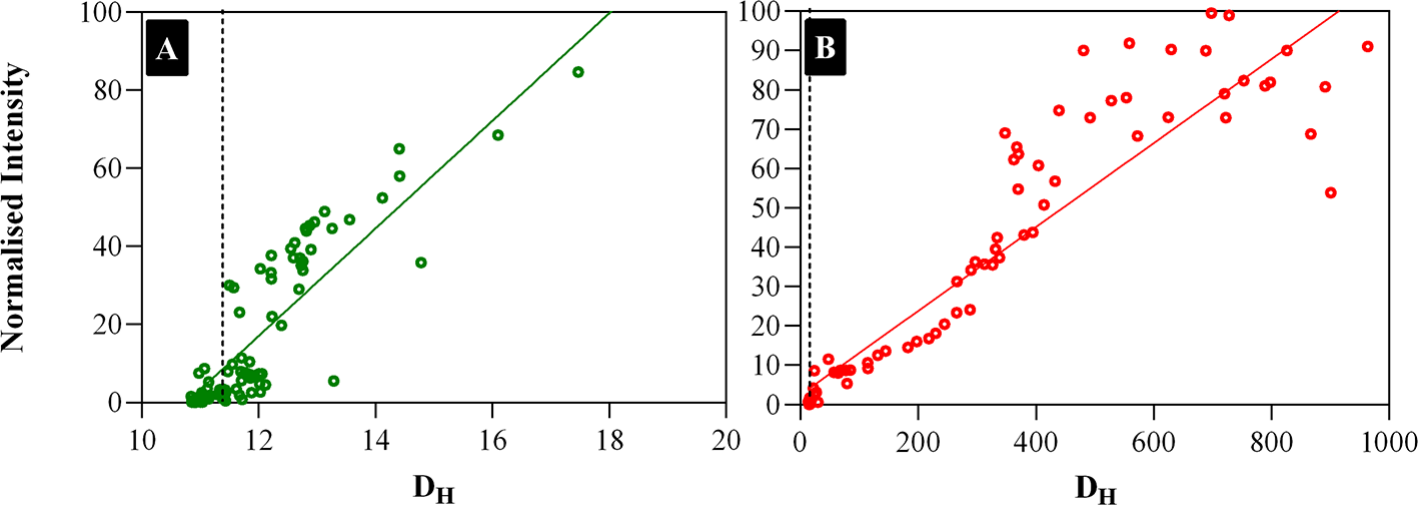
Dependence of hydrodynamic diameter on the intensity of scattered light of 508 ALDHT*t*-native (A) and ALDHT*t*-508 (B). The vertical dotted line corresponds to the value 509 of *D*_H_ at room temperature.

The dependence of hydrodynamic diameter on scattering intensity of ALDH*Tt*-native and ALDH*Tt*-508 during a temperature ramp experiment of 30–70 °C are shown in Figure 8. The relationship between the scattered light intensity and the hydrodynamic diameter is linear. The plots indicate that during heating of ALDH*Tt*-508 to 70 °C, the size of the initial aggregates (i.e., *D*_H_ at which the first increase in light scattering intensity is observed, *D*_H_,_0_) is significantly larger than the size of the initial aggregates produced when heating native ALDH*Tt*. The values of *D*_H,0_ for ALDH*Tt*-508 and ALDH*Tt*-native were determined graphically to be 23.34 and 11.71 nm, respectively. There is a much larger increase in the size of aggregates of ALDH*Tt*-508 than that of ALDH*Tt*-native during heating, with a complete conversion of the mutant protein into aggregates, which can be observed by the shift of the distribution of *D*_H_ to a unimodal population at ∼200 nm (Figure S10). Toward higher temperatures, the *D*_H_ of ALDH*Tt*-508 reached a critical value and begins to drop, leading to the stochastic behavior seen in Figure 8 at *D*_H_ > 400 nm. Figure 8. Dependence of hydrodynamic diameter on the intensity of the scattered light of ALDH*Tt*-native (A) and ALDH*Tt*-508 (B). The vertical dotted line corresponds to the value of *D*_H_ at room temperature.

**Figure 9:**
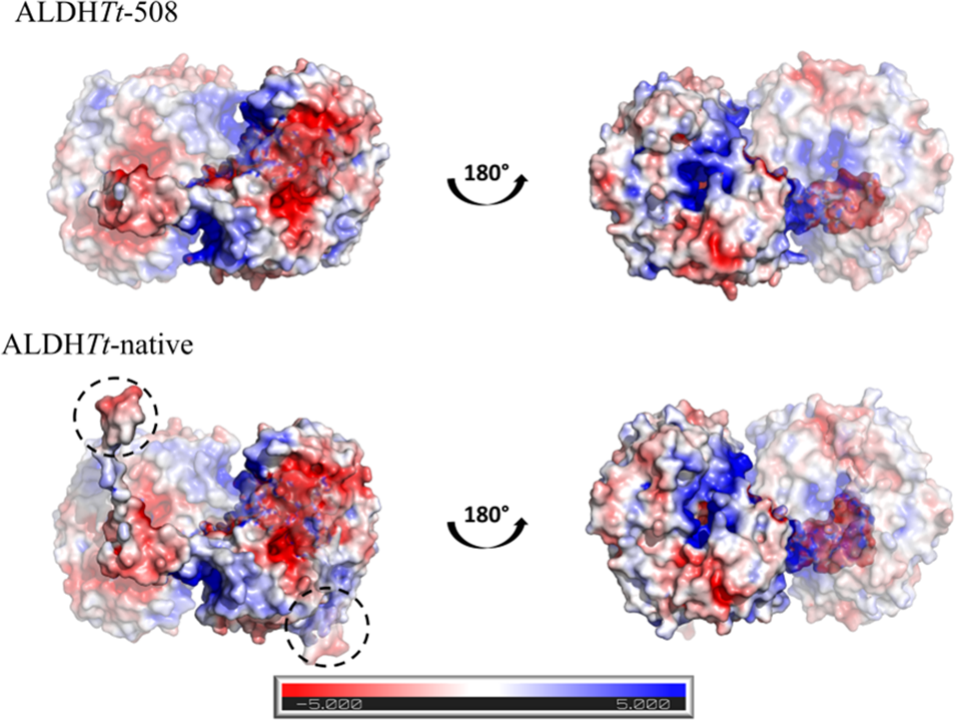
Electrostatics of ALDH*Tt* (dimer) and its truncated mutant, ALDH*Tt*-508. The figure shows the dual polarity of the C-terminal tail (black dashed circle) and the surface charges exposed upon its removal. All ligands from ALDH*Tt*-native and ALDH*Tt*-508 crystallographic data have been removed for clarity (PDB: 6FJX and 6FKV, respectively). Figures were modeled using the Adaptive Poisson–Boltzmann Solver (APBS) available on PyMOL (The PyMOL Molecular Graphics System, Version 2.0 Schrödinger, LLC).^29^

The effect of the truncation on the surface behavior of ALDH*Tt* was visualized by PyMOL (Figure 9). The three-dimensional structure shows that the truncation exposes previously hidden residues that were involved in ionic bonding with the C-terminus, including Ala465, which interacts with Gln510 of the opposing monomer and holds the C-terminus across the aldehyde access tunnel of ALDH*Tt*. The C-terminus can be seen in terminating in a nonpolar, previously described three-turn helix, which does not bond with any of the residues on the surface of the structure, but is however free to move, replicating the movement of previously described “caps” in ALDH active site regulation.^28^ The figure also shows exposure of the highly polar active site, increasing the flexibility of the molecule.

CD spectra representative of both the native ALDH*Tt* protein and ALDH*Tt*-508 are depicted in Figure S11. Both spectra were obtained in the Far-UV region to compare the effect of the missing C-terminal tail on the secondary structure integrity of ALDH*Tt*-508. Both proteins possess negative bands at 208 and 222 cm^–1^, typical of a mostly α-helical protein, in accordance with their crystallographic structure as published on PDB (PDB ID: 6FJX and 6FKV for ALDH*Tt*-native and ALDH*Tt*-511, respectively).^30^ The spectra resemble those published for aldehyde dehydrogenase protein from other sources.^25,31^ The removal of the C-terminus does not seem to have a major effect on the secondary structure as detectable by Far-UV CD. By quantifying the secondary structure composition of both proteins using computational methods, we identified a 5% loss of β-sheet content from the native protein upon C-terminal tail removal and a proportional increase in disordered structures (Table 5). This minor loss could be a result of the partially denatured or aggregated proteins naturally existing in ALDHTt-508 samples, as detectable by SE-HPLC and DLS.^32^ In light of these studies, it is unlikely that the CD data represent a conformational change in the protein upon truncation.

**Table 5:** Quantification of Protein Secondary Structures Using Circular Dichroism Data and the K2D2 Estimation Tool Available on Dichroweb^21,33^

protein	α-helix (%)	β-sheet (%)	random coil (%)
ALDH*Tt*-native	26	21	53
ALDH*Tt*-508	27	16	58

## Discussion

The discovery of a thermophilic aldehyde dehydrogenase from *T. thermophilus* HB27 has led to the elucidation of the role of the C-terminus in ALDH active site control and structural and thermal stability.^15^ An extension of the C-terminus of other bacterial and archaeal thermophilic proteins has been shown to have a vital role in enzymatic behavior and structural integrity.^6,34,35^ Determination of molecular features such as these are vital in exploring the mechanisms of thermostability in proteins and of particular interest to the thermophilic enzyme industry. Crystallographic data of the interactions of the ALDH*Tt*-native C-terminus with the overall quaternary structure suggests that it plays a role in thermostability by introduction of strong ionic and hydrogen bonds across the surface of the homotetramer. Ionic bonds and salt bridges have been identified as some of the most influential contributors to protein thermostability, particularly in exposed protein regions, by decreasing the flexibility of surface residues.^36^ Despite its prominent structure, the extension of the C-terminus has been evolutionarily lost in most ALDH proteins of other thermophilic phylums and of the closely related ALDH from *T. thermophilus* HB8 (Figure 1). In this research, the successful production of a truncated thermostable ALDH protein, ALDH*Tt*-508 allows for the elucidation of C-terminus involvement in ALDH structural stability and aggregation pathways. The size exclusion HPLC studies show that the intrinsic tetrameric structure of ALDH*Tt* is destabilized in mild conditions, leading to a higher propensity toward formation of precipitates when temperature is increased. These results suggest that the C-terminal extension of ALDH*Tt* holds the tetramer together and acts as an oligomerization aid. Many studies of global protein stability report increased rigidity of the biomolecule as a significant marker of thermostability.^2,37,38^ As suggested by the modeling of the surface residues in PyMOL (Figure 9), the removal of the C-terminus increases molecule flexibility by exposing hydrophilic residues at the protein core, causing an increase in the dissociation of the tetramer into dimeric and monomeric species (Table 3).

The production of ALDH*Tt*-508 was optimized to account for its lower thermostability. Heat treatment is routinely used as a simple purification procedure to obtain active, homogeneous thermostable enzymes.^39^ Heat treatment however does not improve yields of the mutant due to effects on the oligomeric state and structural integrity of the protein, as shown in Table 1 and Figure S8. The results from SE-HPLC indicate that loss of the C-terminal extension destabilizes the native tetramer during heat treatment and increases the likelihood of precipitation, most likely by exposure of bond-forming residues and increased intermolecular movement. Similar effect of the C-terminus contribution to oligomeric stability have been recorded in other proteins.^40−42^ Interestingly, heating of the mutant protein in lysate results in the formation of a hexameric species (∼380 kDa), which is likely to be inactive due to the decrease of enzymatic activity in samples subjugated to 15 min lysate treatment (Figure S8, Table 1). These species are most likely to be a result of trimeric and dimeric species present in the sample, with exposed hydrophobic residues prone to intermolecular linkage, prompting aggregation. Plausibly, prolonged exposure of the recombinant protein in *E. coli* lysate provided sufficient time for protein unfolding to occur and subsequent proteolysis of susceptible residues by host cell proteases, which have remained active during thermal treatment.^43,44^

It is clear that removal of the C-terminal extension from ALDH*Tt* affects the protein’s thermophilic properties. Optimisation of the purification process revealed a sensitivity of the mutant enzyme to heat-treatment and displayed improved catalytic activity when the heat-treatment step was minimized. Unlike ALDH*Tt*-native, minimization of the heat-treatment did not lower yields significantly in production of ALDH*Tt*-508.^15,18^ Additionally, monitoring the catalytic activity throughout the purification process revealed a minor increase in the function of ALDH*Tt* after the removal of the C-terminal extension, when both proteins are treated to the same purification method. This result is supported by the data displayed in Table 2, which shows improvement of the kinetics of the enzyme toward the substrate hexanal when the C-terminal extension is removed. This validates the theory that the substrate access tunnel of ALDH*Tt* is obstructed by the 22 amino acid long tail.^15^ The purification profile also suggests that the stability and structure of ALDH*Tt*-508 is retained throughout the purification process, which is performed entirely at room temperature (Table 1). Recombinant ALDH*Tt*-508 therefore holds an economical advantage over production of *E. coli* aldehyde dehydrogenases which are purified at 4 °C.^45^ ALDH*Tt*-508 also holds an economical advantage over ALDH*Tt*-native, due to its improved catalytic activity and shorter purification process. Nickle affinity chromatography alongside a 5 min heat treatment step is sufficient for the homogenization of ALDH*Tt*-508, to a purity of 89.9%. Due to the substantial decrease in yield upon gel filtration, the step is deemed as unnecessary unless a high level of protein purity is needed (i.e., for crystallization) (Table 1).

It is worthy to note that although the dissociation into monomers precedes aggregate formation at raised temperatures, it does not seem to impact negatively on the kinetic behavior of the enzyme at working temperatures of 25 and 50 °C (Table 2). In fact, we report a sharp decrease in the *K*_m_ values for the oxidation of hexanal from 25 to 50 °C, indicating that ALDH*Tt*-508 has a much higher affinity toward the substrate at 50 °C. Surprisingly, the truncated enzyme also shows a much higher affinity toward the substrate than ALDH*Tt*-native, ALDH*Tt-*508, *K*_m_= 0.10 ± 0.01 mM, ALDH*Tt*-native, *K*_m_= 1.98 ± 0.86 at 50 °C. This is possibly explained by the exposure of the catalytic domain, which is present on each monomer, significantly increasing the amount of hexanal binding to the complex per unit of time.^17^ Due to the ability of ALDH*Tt* to oxidize aldehydes using both NAD+ and NADP+ as a cofactor, the kinetics of ALDH*Tt*-508 using NADP+ as a cofactor were studied.^17^ We hypothesized that the opening of the active site would improve the protein’s NADP+ mediated activity due to the exposure of NADP+ binding Glu261, in the cofactor binding cleft. Indeed, our results show that there is a minor increase in the specific enzymatic activity of ALDH*Tt*-508 (0.191 ± 0.034 U/mg) when using NADP+ as a cofactor, compared with ALDH*Tt*-native (0.1453 ± 0.0022 U/mg) (Table 2).

It is expected that the loss of native tetramer structure reflects in a loss of specific enzyme activity per milligram of active enzyme, as reported for other molecules destabilized by C-terminal truncation;^46^ however, this is not the case for ALDH*Tt*-508, as the truncation allows for a more easily accessible active site. This leads to the conclusion that, at the optimum working temperature of the enzyme and in substrate saturated conditions, dissociated dimeric or monomeric species are involved in catalysis or possible reassembly of the native tetramer upon binding of substrate, contributing to its increased enzymatic activity. The formation of precipitates in ALDH*Tt*-508 is temperature-dependent, as evident by the oligomeric stability studies in Table 3, highlighting that the C-terminal tail is paramount in allowing optimum thermostability of the ALDH*Tt*.

The aggregation kinetics of ALDH*Tt* was profoundly altered by the loss of the C-terminus. The destabilization of the molecule at ambient conditions away from the native tetrameric structure induced higher aggregation rates and formation of large insoluble aggregates at 65 and 80 °C (Table 4), as well as accelerating precipitation (Figure 7). This is seen by the higher value of the maximum intensity (*I*_lim_) reached by ALDH*Tt*-508 during heating at both 65° and 80 °C and the lower rate of aggregation (*k*_I_) induced by the presence of the C-terminal extension in ALDH*Tt*-native. This result is easily explainable by the increased mobility of the enzyme upon C-terminal truncation, leading to increased intramolecular interaction.^47^ The increased mobility is induced by the exposure of positively charged hydrophilic regions present in the substrate access tunnel (Figure 9). The aggregation behavior of human ALDHs and their contribution to disease is well studied; however, there are no studies reported on the aggregation kinetics of thermophilic aldehyde dehydrogenases.^17^ The homotetrameric ALDH, rabbit muscle glyceraldehyde-3-phosphate dehydrogenase (GAPDH), follows a similar exponential mechanism of aggregation to ALDH*Tt,* with a critical point of precipitation.^48,49^ Unlike GAPDH, we found no evidence of tetramer dissociation of ALDH*Tt*-native at elevated temperatures during aggregation kinetic studies, and the aggregates formed can be assumed to constitute unfolded or partially unfolded intact tetramers. Rabbit muscle GAPDH is a mesophilic enzyme with no structural evidence of an oligomer anchoring tail, such as the C-terminus in ALDH*Tt*, which could explain its dissociative aggregation mechanism^50^ ALDH*Tt*-508 dissociation into dimeric and monomeric species was inherent to the protein at ambient temperatures. SE-HPLC data dictate that lower molecular weight species are involved during the aggregation and precipitation of ALDH*Tt*-508 (Table 3).

Despite the destabilization of the tetramer by the truncation, the research within this paper also showcases why the C-terminal extension has been evolutionarily excised from ALDH proteins in other organisms. Truncation of the protein by 22 amino acids caused a full opening of the substrate tunnel by the removal of residue Q510, thus severing the bond between the C-terminal and the substrate anchoring region. This is apparent by the substantial increase in activity after purification of ALDH*Tt*-508 (Table 1) and by the large increase in activity at all pH and temperatures tested, when compared with the literature (Figure 4^18^). The loss in thermostability indicated by the previously determined *T*_m_ (ALDH*Tt*-native *T*_m_ = 84 °C, ALDH*Tt*-511 *T*_m_ = 80 °C) is not reflected in the temperature profile trialled during this study, as the protein is fully active at highest temperature compatible with the aldehyde substrates (50 °C) (Figures 4A and 5). Opening of the homotetramer complex by removal of the C-terminus also increased the substrate specificity toward aldehydes of ALDH*Tt.* In particular, the truncated enzyme gained the ability to catalyze reactions with large and/or cyclic aldehydes such as furfural, citral, *o*-tolualdehyde, and cyclohexaneboroaldehyde. This can also be explained by the opening of the substrate tunnel upon C-terminus removal, enhancing accessibility of the catalytic thiol to large molecules.

The blocking of the active site by the native C-terminal tail is apparent at all pH levels, as pH profiles reported on ALDH*Tt* show a lower specific enzyme activity of the protein in pH buffers 2–10.^18^ In fact, ALDH*Tt*-508 performs significantly better at lower pH values, with a 2-fold increase in activity at pH 6 and 7 and a 3-fold increase in activity at pH 5. Additionally, mutant enzyme ALDHTt-508 is active at low-pH citrate buffers, pH 3 and 4, whereas ALDH*Tt*-native shows no reported activity at pH 2–4.^18^ The truncation of the C-terminus seems to expand the protein’s stability toward acidic pH environments (Figure 4B). This result was unexpected due to the apparent loss of stabilizing salt bridges and H-bonds from the structure after the mutation. Other enzymes originating from the *T. thermophilus* bacterium are also prone to activity loss at acidic pHs, making the mutant unique in its pH profile.^51,52^ One explanation for the apparent improvement in catalytic efficiency at low pH could be the exposure of negatively charged surface residues which previously interacted with the C-terminal extension, altering the overall surface charge of the protein.^53,54^ This is demonstrated by the analysis of the surface charges calculated by using the APBS (Adaptive Poisson–Boltzmann Solver) software available in PyMOL (Figure 9). Although an excess of net charges may decrease the overall conformational stability of protein molecules, it has been shown to decrease aggregation rates by electrostatic repulsion between surface charges.^24^

The finding implies that the mutant ALDH*Tt*-508 is a better contender for applications in aldehyde and alcohol catalysis reactions which require an acidic pH. Further investigation is needed for the protein’s stability over time at acidic pH, as the aggregation kinetics studied within this paper were conducted only at pH 8.0. Under acidic conditions, the effect of electrostatic repulsion may improve the aggregation kinetics reported in this study.

In conclusion, an ALDH from *Thermus thermophilus* was successfully produced without its native C-terminal extension to probe the impact of the truncation on substrate affinity, pH and temperature stability, oligomeric stability, and aggregation kinetics. Results obtained in this study testify that the removal of the C-terminus broadens the substrate specificity of the enzyme to include a broader range of cyclic/large aldehydes by opening of the active site. The oxidoreductase activity of ALDH*Tt* is substantially increased at acidic pH values by the exposure of negative surface charges. The results herein show that the kinetics of ALDH*Tt* aggregation do not involve the formation of stable intermediates or dissociated states and therefore follow a monomolecular reaction. The C-terminal extension is also shown to increase molecular rigidity by shielding polar residues involved in catalysis.

## Supporting Information Available

Additional figures demonstrate the update for PDB FASTA sequence entry: 6FKV, for protein ALDH*Tt*-508 (UniProtKB Q72KD3) (Figure S1); elution profiles of ALDH*Tt*-508 and ALDH*Tt*-native during the purification process (Figures S2 and S3, respectively); MALDI-TOF spectra of ALDH*Tt*-508, confirming the monomeric molecular weight of 57,055.97 kDa (Figure S4); Michealis–Menten kinetics of ALDH*Tt*-508 (Figures S5 and S6), showcasing its affinity to hexanal using NAD+ or NADP+ as a cofactor, respectively; relative activity of the substrate affinities of ALDHTt-native and ALDHTt-508 with respect to the substrate hexanal (Figure S7); calibration of size-exclusion columns and elution profiles during heat-shock optimization (Figure S8); size distribution of the hydrodynamic diameter (*D*_H_) of ALDH*Tt*-508 at 25 °C (Figure S9) and during a heat-ramp experiment (Figure S10); and circular dichroism data used to quantify ALDH*Tt*-native and ALDH*Tt*-508 secondary structure composition (Figure S11) (PDF)
