# Supplementary material for: Contribution of a C-Terminal Extension to the Substrate Affinity and Oligomeric Stability of Aldehyde Dehydrogenase from Thermus thermophilus HB27
Source: Biochemistry. 2024 Apr 11;63(9):1075–88. doi: 10.1021/acs.biochem.3c00698 (PMC11080044; doi:10.1021/acs.biochem.3c00698)
Supplement: Supplementary file 1 — bi3c00698_si_001.pdf [file bi3c00698_si_001.pdf]

Contribution of a C-terminal extension to the substrate affinity and oligomeric stability of aldehyde dehydrogenase from *T. thermophilus* HB27

Wiktoria Brytan [a], Kim Shortall [a], Francisco Duarte [a], Tewfik Soulimane* [a, b], Luis Padrela* [a, b]

[a] Department of Chemical Sciences, Bernal Institute, University of Limerick, Limerick, Ireland

[b] SSPC – The Science Foundation Ireland Research Centre for Pharmaceuticals, Ireland

*Corresponding authors email addresses: Tewfik.Soulimane@ul.ie; Luis.Padrela@ul.ie

Supporting information

FIGURE LEGEND

Figure S1. Left: Sequence of ALDH*Tt*-511 available on PBD (PDB entry: 6FKV) deposited under ALDH*Tt*-508. Right: sequence of ALDH*Tt*-508 produced in this publication and (18). Both proteins are expressed with 6x-Histidine tag which is retained after the production process

Figure S2. Elution of ALDH*Tt*-508 during A) Nickle-affinity chromatography, and B) Size-exclusion chromatography. Figure 3A) shows the elution of ALDH*Tt*-508 at 20 % Imidazole (200 mM) with a secondary elution peak at 50 % (500mM) imidazole. The presence of ALDHTt-508 in these fractions was confirmed by 12% SDS-PAGE (below). Figure 3B) shows the elution of ALDH*Tt*-508, treated with 5 min heat shock of cell lysate, from a HiLoad 16/60 Superdex 200 pg size exclusion chromatography column.

Figure S3. Elution profiles of ALDH*Tt*-native using Nickle affinity (A) and gel filtration (B).

Figure S4. MALDI-TOF spectrum of ALDH*Tt*-508 with truncated C-terminal tail.

Figure S5. Michaelis-Menten Kinetics of ALDH*Tt*-508 using the aldehyde hexanal as substrate at A) 25 °C and B) 50 °C. Curves were fitted using OriginPro 2023.

Figure S6. Michaelis-Menten Kinetics of ALDH*Tt*-508 using the aldehyde hexanal as substrate and NADP as a cofactor at 50 °C. Curves were fitted using GraphPad Prism (Ver.10.0.1).

Figure S7. Relative activity of ALDH*Tt*-native and ALDH*Tt*-508 towards various aldehydes, with respect to the model substrate hexanal, at 25°C and 50°C.

Figure S8. SE-HPLC signal detected at 280 nm for the ALDH*Tt*-508 protein after subjecting the lysate to three levels of heat treatment at 65 °C (A). The mutant protein eluted at ~5.6 min. The molecular weight of the protein and its oligomeric states was confirmed by a calibration curve using four molecules of known molecular weight (B).

Figure S9. Size distribution by intensity of the hydrodynamic diameter of ALDH*Tt*-508, measured in 10 mM KPO_4 buffer at 25 °C. The data of all triplicates are shown.

Figure S10. Frequency distribution by volume of ALDH*Tt*-508 during a ramp temperature experiment from 25 °C to 80 °C in 10mM potassium phosphate buffer.

Figure S11. Effect of truncation of the C-terminus extension on the ALDHTt protein's intrinsic secondary structure. ALDH*Tt*-508 (red) and ALDH*Tt*- native (green). The spectra were obtained in 0.005 mg/ml protein concentration in 10 mM potassium phosphate buffer, pH 8.0. The data is expressed in units of theta (degcm^2/dmol). The spectra were analysed using ProData Chirascan software.

Figure S1

```
>ALDHTt-511, PDB no.: 6FKV_1 |Aldehyde dehydrogenase | Thermus thermophilus HB27 (262724)
10 20 30 40 50 60
MHHHHHHRKA AGKYGNTLEF GHLVGGEEVL EGPLLERRNP SDREDVVARF PEADKDLVRK

70 80 90 100 110 120
AALKAREAFA EWSRTPAPIR GQVLFNLVKI LEREKPTLTR LMVREVGKTP KEAAGDVQEA

130 140 150 160 170 180
IDTALFFASE GRRLYGQTVP SEMRDKELFT FRRPLGVVGI ITAGNFPIAV PSWKLIPAVL

190 200 210 220 230 240
TGNTVVWKPS EDAPTLSFVF AKLFEEAGLP PGVLNVVFGG GKGSTGQWMV ELMDEGLFQK

250 260 270 280 290 300
FAFTGSTQVG RWIGEVAGRN LIRPTLELGG KNPLVVMRDA DLDLAVEGAW WSAFATGGQR

310 320 330 340 350 360
CTSAGNILVD APIYEEFKRR FLERVEATLV GNPLLHPEVT YGPFINERFF ARWQEHYRVG

370 380 390 400 410 420
EAEGARLLFG RGRITRENPY PRFLGDPEAG LYGWPTVWEV RPGTRLFTEE VFGPTINLVK

430 440 450 460 470 480
VDGIEEAIAV ANSTPYGLSS AIYTNHRHWA YLFKVGIRAG MTSINNATVG AEAHLPFGGV

490 500 510
KASGNGGRES GIWVLEEYTY WHAVNEEYSG RLQLAQM
```

```
>ALDHTt-508 |Aldehyde dehydrogenase | Thermus thermophilus HB27
10 20 30 40 50 60
MHHHHHHRKA AGKYGNTLEF GHLVGGEEVL EGPLLERRNP SDREDVVARF PEADKDLVRK

70 80 90 100 110 120
AALKAREAFA EWSRTPAPIR GQVLFNLVKI LEREKPTLTR LMVREVGKTP KEAAGDVQEA

130 140 150 160 170 180
IDTALFFASE GRRLYGQTVP SEMRDKELFT FRRPLGVVGI ITAGNFPIAV PSWKLIPAVL

190 200 210 220 230 240
TGNTVVWKPS EDAPTLSFVF AKLFEEAGLP PGVLNVVFGG GKGSTGQWMV ELMDEGLFQK

250 260 270 280 290 300
FAFTGSTQVG RWIGEVAGRN LIRPTLELGG KNPLVVMRDA DLDLAVEGAW WSAFATGGQR

310 320 330 340 350 360
CTSAGNILVD APIYEEFKRR FLERVEATLV GNPLLHPEVT YGPFINERFF ARWQEHYRVG

370 380 390 400 410 420
EAEGARLLFG RGRITRENPY PRFLGDPEAG LYGWPTVWEV RPGTRLFTEE VFGPTINLVK

430 440 450 460 470 480
VDGIEEAIAV ANSTPYGLSS AIYTNHRHWA YLFKVGIRAG MTSINNATVG AEAHLPFGGV

490 500 510
KASGNGGRES GIWVLEEYTY WHAVNEEYSG RLQL
```

Figure S2

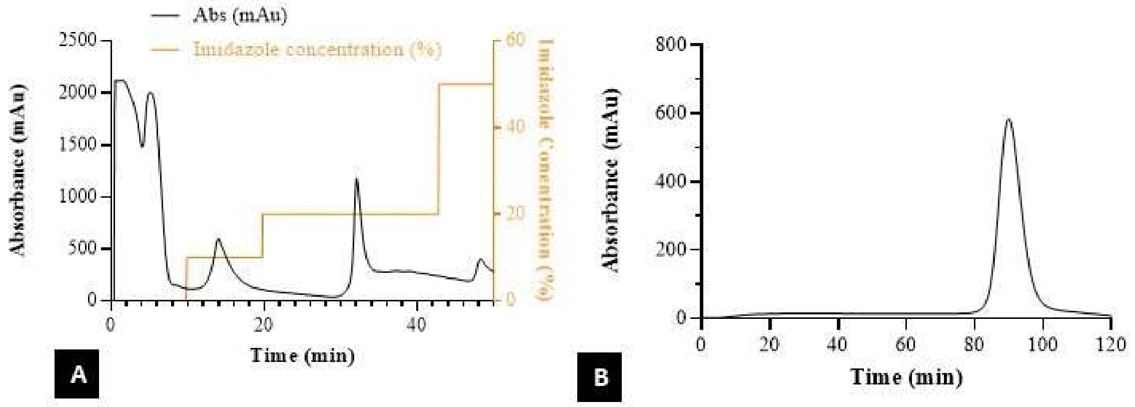

Figure S3

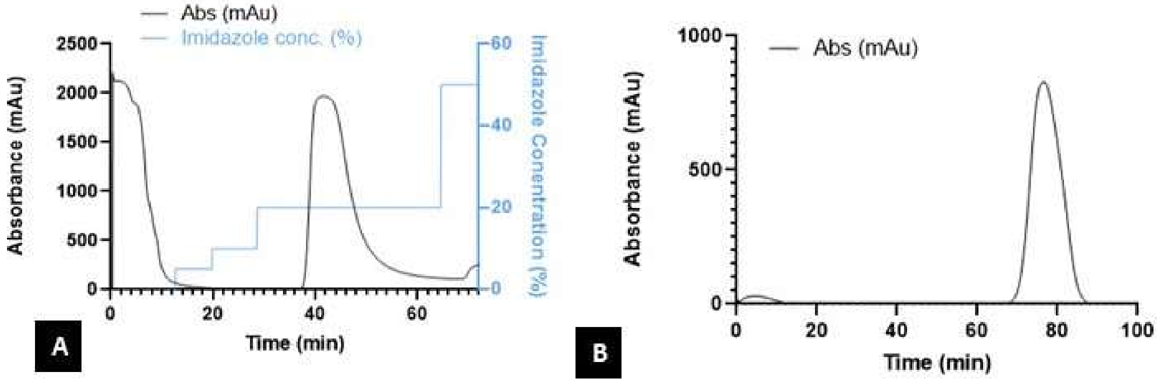

Figure S4

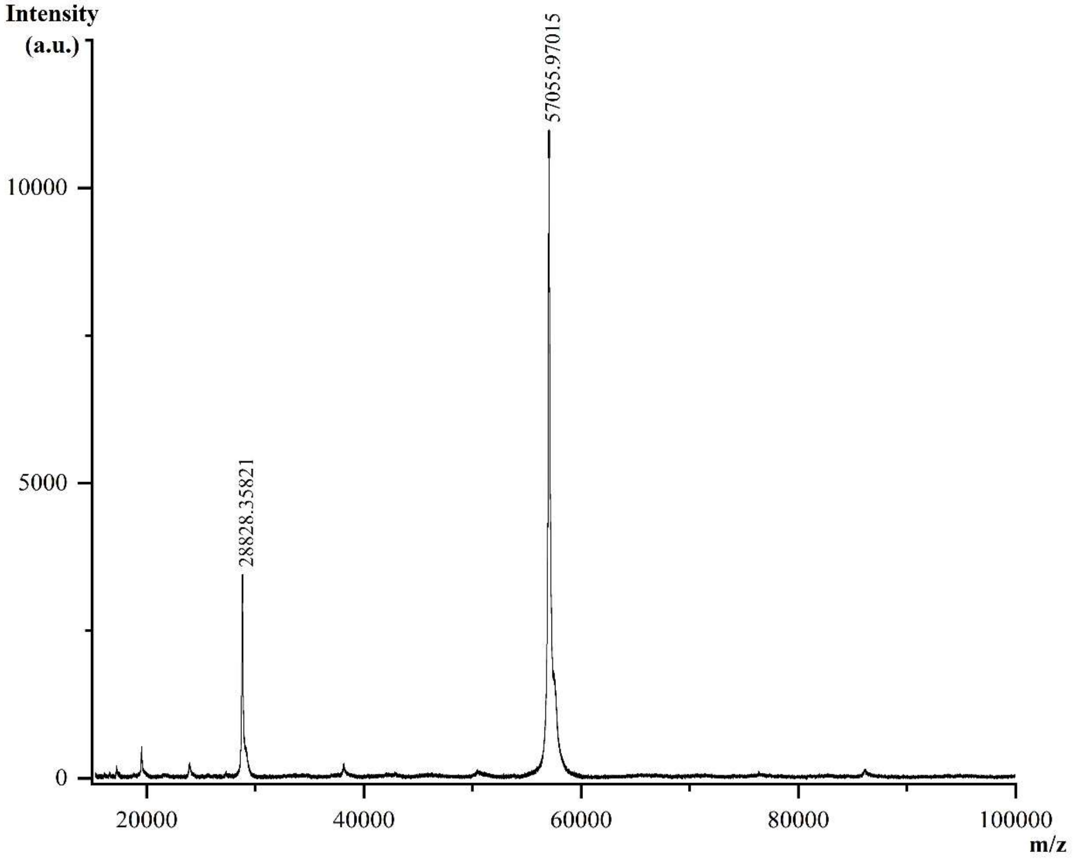
Intensity
(a.u.)
10000
5000
0
57055.97015
28828.35821
20000
40000
60000
80000
100000
m/z

Figure S5

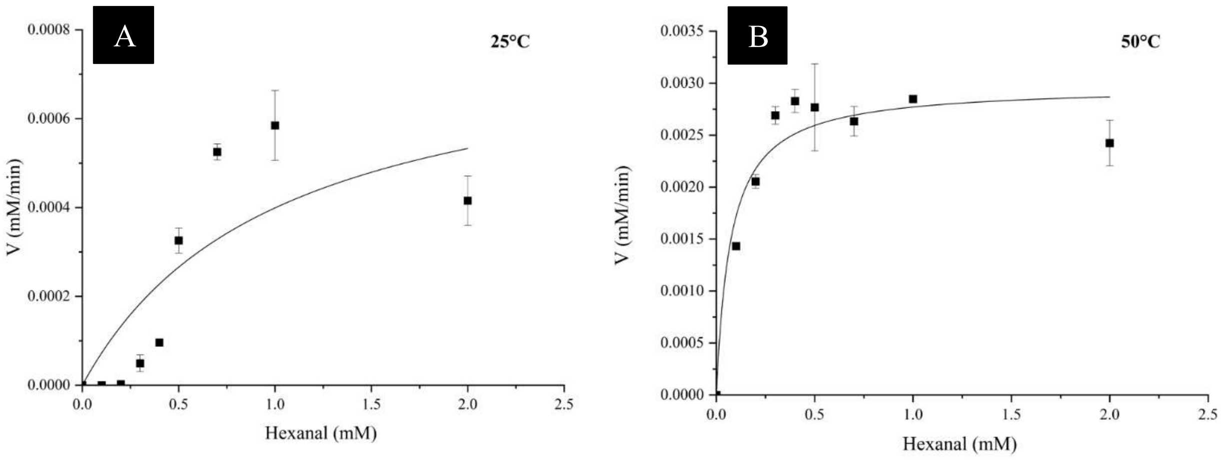

Figure S6

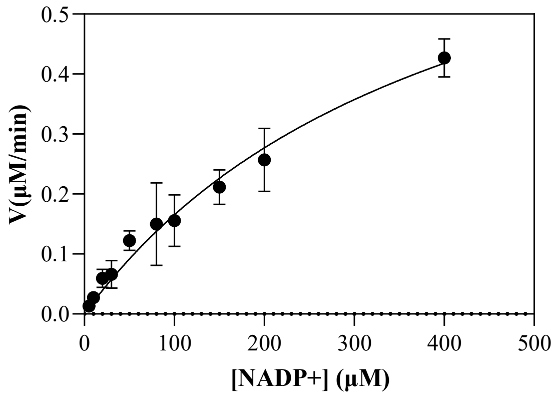

Figure S7

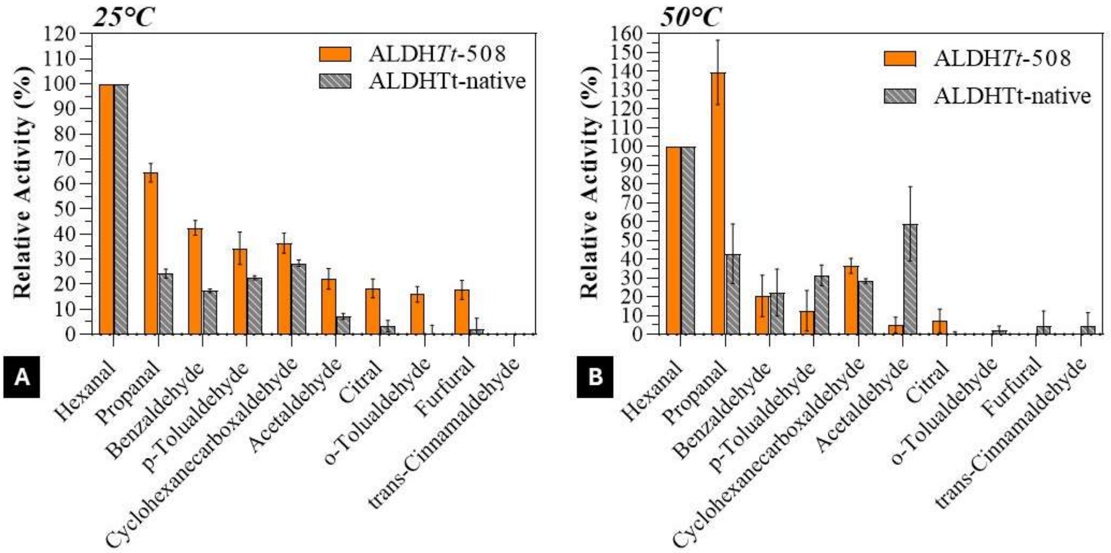
25°C
50°C
Relative Activity (%)
ALDHTt-508
ALDHTt-native
A
B
Hexanal
Propanal
Benzaldehyde
p-Tolualdehyde
Cyclohexanecarboxaldehyde
Acetaldehyde
Citral
o-Tolualdehyde
Furfural
trans-Cinnamaldehyde

Figure S8

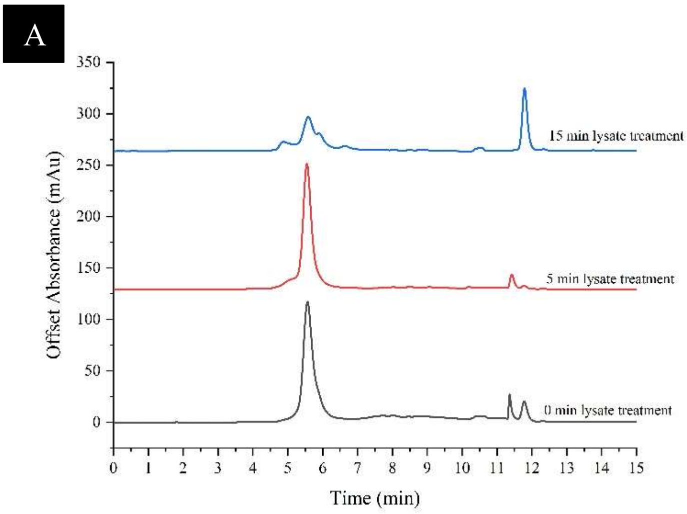
A
350
300
250
200
150
100
50
0
Offset Absorbance (mAu)
15 min lysate treatment
5 min lysate treatment
0 min lysate treatment
0 1 2 3 4 5 6 7 8 9 10 11 12 13 14 15
Time (min)

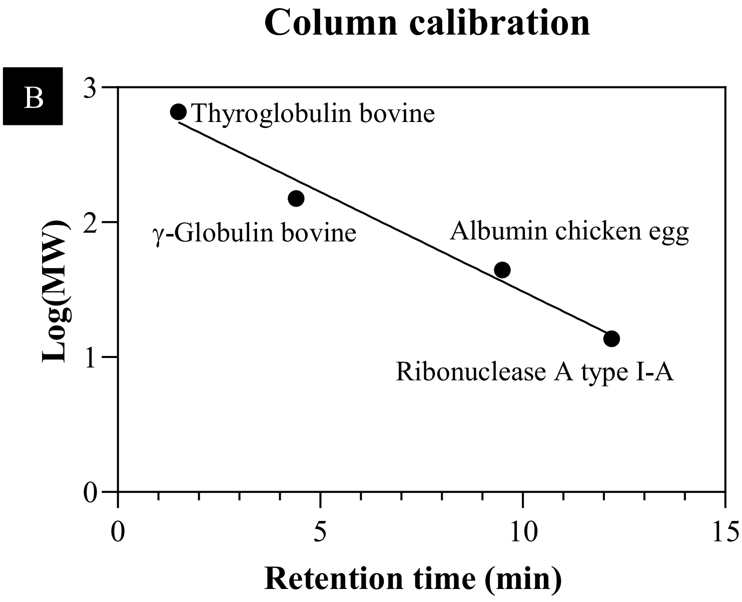
Column calibration
B
3
2
1
0
Log(MW)
Thyroglobulin bovine
γ-Globulin bovine
Albumin chicken egg
Ribonuclease A type I-A
0 5 10 15
Retention time (min)

Figure S9

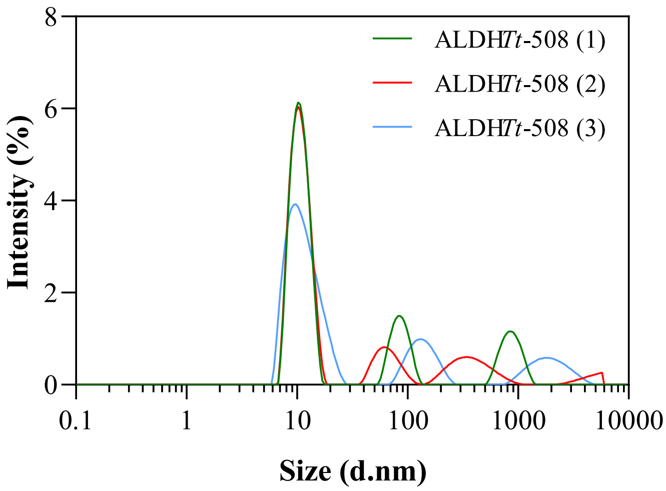

Figure S10

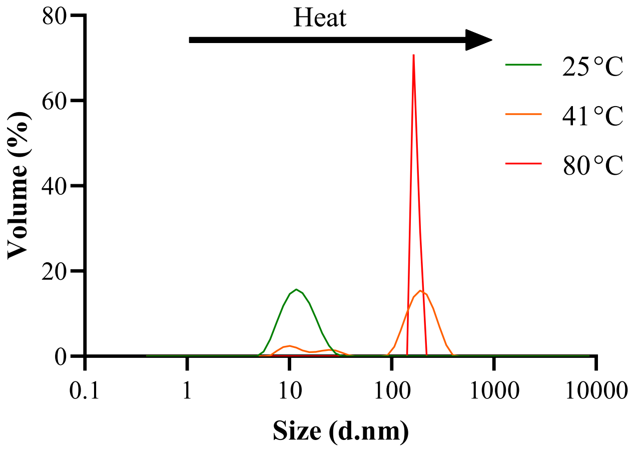

Figure S11

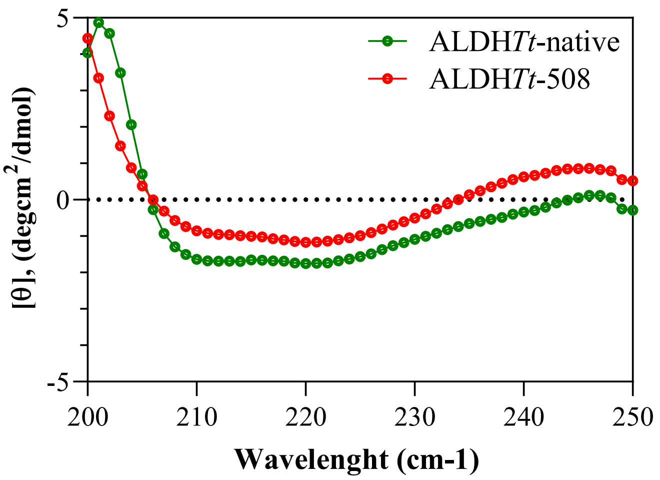
ALDHTt-native
ALDHTt-508
[θ], (degcm2/dmol)
5
0
-5
200
210
220
230
240
250
Wavelenght (cm-1)
